# Clinical characteristics, surveillance, treatment allocation, and outcomes of non-alcoholic fatty liver disease-related hepatocellular carcinoma: a systematic review and meta-analysis

**DOI:** 10.1016/S1470-2045(22)00078-X

**Published:** 2022-03-04

**Authors:** Darren Jun Hao Tan, Cheng Han Ng, Snow Yunni Lin, Xin Hui Pan, Phoebe Tay, Wen Hui Lim, Margaret Teng, Nicholas Syn, Grace Lim, Jie Ning Yong, Jingxuan Quek, Jieling Xiao, Yock Young Dan, Mohammad Shadab Siddiqui, Arun J Sanyal, Mark D Muthiah, Rohit Loomba, Daniel Q Huang

**Affiliations:** Department of Medicine, Yong Loo Lin School of Medicine, National University of Singapore, Singapore (D J H Tan, C H Ng, S Y Lin, X H Pan, P Tay, W H Lim, N Syn MBBS, J N Yong, J Quek, J Xiao, Prof Y Y Dan MBBS, M D Muthiah MBBS, D Q Huang MBBS); Division of Gastroenterology and Hepatology, Department of Medicine, National University Hospital, Singapore (M Teng MBBS, Prof Y Y Dan, M D Muthiah, D Q Huang); Lee Kong Chian School of Medicine, Nanyang Technological University, Singapore (G Lim); Division of Gastroenterology, Hepatology, and Nutrition, Department of Internal Medicine, Virginia Commonwealth University, Richmond, VA, USA (M S Siddiqui MD, Prof A J Sanyal MD); National University Centre for Organ Transplantation, National University Health System, Singapore (M D Muthiah, D Q Huang); NAFLD Research Center, Division of Gastroenterology, University of California at San Diego, La Jolla, CA, USA (Prof R Loomba MD, D Q Huang)

## Abstract

**Background:**

The clinical presentation and outcomes of non-alcoholic fatty liver disease (NAFLD)-related hepatocellular carcinoma are unclear when compared with hepatocellular carcinoma due to other causes. We aimed to establish the prevalence, clinical features, surveillance rates, treatment allocation, and outcomes of NAFLD-related hepatocellular carcinoma.

**Methods:**

In this systematic review and meta-analysis, we searched MEDLINE and Embase from inception until Jan 17, 2022, for articles in English that compared clinical features, and outcomes of NAFLD-related hepatocellular carcinoma versus hepatocellular carcinoma due to other causes. We included cross-sectional and longitudinal observational studies and excluded paediatric studies. Study-level data were extracted from the published reports. The primary outcomes were (1) the proportion of hepatocellular carcinoma secondary to NAFLD, (2) comparison of patient and tumour characteristics of NAFLD-related hepatocellular carcinoma versus other causes, and (3) comparison of surveillance, treatment allocation, and overall and disease-free survival outcomes of NAFLD-related versus non-NAFLD-related hepatocellular carcinoma. We analysed proportional data using a generalised linear mixed model. Pairwise meta-analysis was done to obtain odds ratio (OR) or mean difference, comparing NAFLD-related with non-NAFLD-related hepatocellular carcinoma. We evaluated survival outcomes using pooled analysis of hazard ratios.

**Findings:**

Of 3631 records identified, 61 studies (done between January, 1980, and May, 2021; 94 636 patients) met inclusion criteria. Overall, the proportion of hepatocellular carcinoma cases secondary to NAFLD was 15·1% (95% CI 11·9–18·9). Patients with NAFLD-related hepatocellular carcinoma were older (p<0·0001), had higher BMI (p<0·0001), and were more likely to present with metabolic comorbidities (diabetes [p<0·0001], hypertension [p<0·0001], and hyperlipidaemia [p<0·0001]) or cardiovascular disease at presentation (p=0·0055) than patients with hepatocellular carcinoma due to other causes. They were also more likely to be non-cirrhotic (38·5%, 27·9–50·2 *vs* 14·6%, 8·7–23·4 for hepatocellular carcinoma due to other causes; p<0·0001). Patients with NAFLD-related hepatocellular carcinoma had larger tumour diameters (p=0·0087), were more likely to have uninodular lesions (p=0·0003), and had similar odds of Barcelona Clinic Liver Cancer stages, TNM stages, alpha fetoprotein concentration, and Eastern Cooperative Oncology Group (ECOG) performance status to patients with non-NAFLD-related hepatocellular carcinoma. A lower proportion of patients with NAFLD-related hepatocellular carcinoma underwent surveillance (32·8%, 12·0–63·7) than did patients with hepatocellular carcinoma due to other causes (55·7%, 24·0–83·3; p<0·0001). There were no significant differences in treatment allocation (curative therapy, palliative therapy, and best supportive care) between patients with NAFLD-related hepatocellular carcinoma and those with hepatocellular carcinoma due to other causes. Overall survival did not differ between the two groups (hazard ratio 1·05, 95% CI 0·92–1·20, p=0·43), but disease-free survival was longer for patients with NAFLD-related hepatocellular carcinoma (0·79, 0·63–0·99; p=0·044). There was substantial heterogeneity in most analyses (*I*^2^>75%), and all articles had low-to-moderate risk of bias.

**Interpretation:**

NAFLD-related hepatocellular carcinoma is associated with a higher proportion of patients without cirrhosis and lower surveillance rates than hepatocellular carcinoma due to other causes. Surveillance strategies should be developed for patients with NAFLD without cirrhosis who are at high risk of developing hepatocellular carcinoma.

**Funding:**

None.

## Editorial note

the *Lancet* Group takes a neutral position with respect to territorial claims in published maps and institutional affiliations.

## Introduction

Hepatocellular carcinoma is the third leading cause of cancer-related deaths worldwide.^1,2^ With recent advances in the treatment of hepatitis B virus (HBV) and hepatitis C virus (HCV) infection, the burden of hepatocellular carcinoma due to viral hepatitis is declining; however, the prevalence of non-alcoholic fatty liver disease (NAFLD)-related hepatocellular carcinoma is rising rapidly.^3,4^ Nearly a third of the global population has NAFLD, of whom around 20% harbour non-alcoholic steatohepatitis, which can progress to cirrhosis and hepatocellular carcinoma.^5–8^ NAFLD is the fastest rising cause of hepatocellular carcinoma in the USA and parts of Europe, and is expected to rise exponentially in parallel with the global obesity epidemic.^9–12^

However, data regarding the clinical presentation and outcomes for NAFLD-related hepatocellular carcinoma versus hepatocellular carcinoma due to other causes are conflicted. Several studies reported more advanced disease at presentation and poorer survival among patients with NAFLD-related hepatocellular carcinoma compared with those with hepatocellular carcinoma due to other causes, whereas other studies have reported similar clinical presentation and improved survival.^13–15^ In addition, it is unclear what proportion of patients with NAFLD-related hepatocellular carcinoma do not have cirrhosis or receive surveillance before a hepatocellular carcinoma diagnosis. Previous studies on this topic have been specific to a country, region, or treatment. To our knowledge, a comprehensive meta-analysis evaluating the clinical presentation, proportion without cirrhosis, surveillance rates, and overall outcomes between NAFLD-related and non-NAFLD-related hepatocellular carcinoma has not been reported. Therefore, through a systematic review and meta-analytic approach, we aimed to identify the clinical features, surveillance rates, treatment allocation, and survival outcomes of NAFLD-related hepatocellular carcinoma compared with hepatocellular carcinoma due to other causes.

## Methods

### Search strategy and selection criteria

This systematic review and meta-analysis adhered to the Preferred Reporting Items for Systematic Reviews and Meta-Analyses for its synthesis.^16^ MEDLINE and Embase were searched for articles describing prevalence, risk factors, and outcomes of NAFLD-related hepatocellular carcinoma versus hepatocellular carcinoma due to other causes from inception to Jan 17, 2022. Key search terms included “non-alcoholic fatty liver” and “hepatocellular carcinoma”. The full search strategy can be found in the appendix (p 1). To ensure a comprehensive search, we screened for grey literature by reviewing the bibliographies of included articles and previous meta-analyses.

Four authors (DJHT, SYL, PT, and XHP) independently filtered the title abstracts, followed by full-text review. Discrepancies were resolved by consensus or in consultation with a senior author (DQH). Original articles, including prospective and retrospective cohort studies and randomised control trials, were considered for inclusion whereas we excluded reviews, commentaries, and editorials. We included studies written or translated into English. Studies were included if they reported clinical characteristics, risk factors, screening, treatment allocation, or survival outcomes of patients diagnosed with hepatocellular carcinoma, and compared these characteristics between NAFLD-related versus non-NAFLD-related hepatocellular carcinoma. Studies done in the paediatric population, and studies focusing on hepatocellular carcinoma secondary to cryptogenic liver disease were excluded, unless the authors of the study specified that the cryptogenic liver disease cases were probably due to NAFLD. We did not include data from unpublished studies and trial registries up to Jan 17, 2022, in the pooled analysis. For multiple studies inferring results from the same databases, we removed overlapping studies and only included the most updated studies. The protocol is available in the appendix (pp 29–31).

### Data analysis

NAFLD was defined based on either imaging, histology, or International Classification of Diseases codes in the absence of significant alcohol consumption and coexisting causes of chronic liver disease. Hepatocellular carcinoma was diagnosed either on the basis of histology, regardless of subtypes, or by imaging (including CT, MRI, and contrast-enhanced ultrasound). Surveillance for hepatocellular carcinoma was defined as bi-annual imaging of the liver via ultrasound scan, CT or MRI, with or without measurement of serum alpha fetoprotein.^17^ Treatment methods were classified into curative therapies, palliative therapies, and best supportive care. Curative therapies included liver transplantation, liver resection, and ablation. Palliative therapies included transarterial chemoembolisation, radioembolisation, radiotherapy, and systemic therapy. Six authors (DJHT, SYL, CHN, XHP, PT, and WHL) independently extracted study-level aggregated data including study characteristics (author, country, and study design), patient characteristics (age, sex, presence of cirrhosis, diagnostic criteria, body-mass index [BMI], presence of metabolic conditions, and Eastern Cooperative Oncology Group [ECOG] status), tumour characteristics (Barcelona Clinic Liver Cancer [BCLC] staging, TNM staging, tumour diameter, and serum alpha fetoprotein), treatment methods, whether patients underwent surveillance before diagnosis of hepatocellular carcinoma, and survival outcomes (overall survival and disease-free survival). Overall survival was defined as the duration from the date of hepatocellular carcinoma diagnosis to date of death by any cause. Disease-free survival was defined as the duration from date of definitive curative treatment to date of disease recurrence or death. We extracted survival data via reconstruction of published Kaplan-Meier curves using the method by Guyot and colleagues,^18^ which remains the gold standard in survival analysis.^19–21^ During the data extraction process, for articles that only provided data in the form of median (IQR) and not mean (SD), we transformed values using pre-existing formulae, where mean and SDs were estimated from median and IQR with the widely adopted formulas by Wan and colleagues.^22^

### Outcomes

The primary outcomes of the study were (1) the proportion of hepatocellular carcinoma secondary to NAFLD globally, with prespecified subgroup analysis by WHO region (European Region, Region of the Americas, South-East Asian Region, African region, Eastern Mediterranean region, and the Western Pacific Region) and over time (before 2000; 2000–04; 2005–09; 2010, and beyond); (2) comparison of patient and tumour characteristics of NAFLD-related hepatocellular carcinoma versus hepatocellular carcinoma due to other causes; and (3) comparison of surveillance, treatment allocation (curative treatment, palliative treatments, or best supportive care), and survival outcomes of NAFLD-related hepatocellular carcinoma versus other causes. We performed prespecified subgroup analyses by individual causes of liver disease (HBV, HCV, and alcohol-associated liver disease) for comparison of patient and tumour characteristics, surveillance, treatment allocation, and survival outcomes. Subgroup analysis was also done for the proportion of patients with NAFLD-related hepatocellular carcinoma receiving each treatment type (curative *vs* palliative treatment), and for the specific type of curative treatment (liver transplantation, liver resection, or ablation). For analysis of survival outcomes, prespecified sensitivity analyses were conducted in patients with cirrhosis, in patients receiving all types of curative treatment, and by type of curative treatment (liver transplantation, liver resection, and ablation).

### Statistical analysis

Statistical heterogeneity for the pooled estimates was assessed via *I*^2^ and Cochran’s Q test values, where an *I*^2^ value of 25% represented a low degree of heterogeneity, 50% represented a moderate degree, and 75% represented a high degree.^23,24^

We used a random effects model in all analyses regardless of heterogeneity measures as evidence has shown more robust effect estimates compared with fixed effect models.^25,26^ Meta-analysis of proportions was done with a generalised linear mixed model with Clopper-Pearson intervals to estimate the overall proportion and corresponding 95% CI of hepatocellular carcinoma secondary to NAFLD and for the prespecified subgroup analysis by WHO region and time periods.^27,28^ Post-hoc subgroup analysis for proportion of hepatocellular carcinoma secondary to NAFLD was performed by clinical cohort versus administrative database studies with similar methods to the primary analysis.

Differences in patient and tumour characteristics, surveillance, and treatment allocation between NAFLD-related hepatocellular carcinoma and hepatocellular carcinoma due to other causes for both prespecified subgroup analyses and post-hoc analyses were evaluated via a comparative meta-analysis in odds ratios (OR) for binary variables, and mean difference for continuous variables with the DerSimonian and Laird random effects model.^24^ Additional post-hoc subgroup analyses of patient and tumour characteristics in NAFLD-related hepatocellular carcinoma versus other causes were further stratified by cause of hepatocellular carcinoma (HBV, HCV, and alcohol) and WHO region, and analysed with the DerSimonian and Laird random effects model.

For survival outcomes (overall survival and disease-free survival) and in prespecified sensitivity analysis, pairwise analysis was conducted with hazard ratios (HR) with the DerSimonian and Laird random effects model to compare between NAFLD-related and non-NAFLD-related hepatocellular carcinoma. Prespecified meta-regression was considered for the adjustment of baseline characteristics on treatment allocation and survival outcomes.^29,30^

We used RStudio (version 1.3.1093) for all analyses. A two-tailed p value of less than 0·05 was considered as the threshold for statistical significance.

We used the Joanna Briggs Institute (JBI) Critical Appraisal Tool for quality assessment of included articles.^31^ The JBI assessment rates the risk of bias of cohort studies on the basis of appropriateness of sample frame, sampling method, adequacy of sample size, data analysis, methods for identification and measurement of relevant conditions, statistical analysis, and response rate adequacy. We assessed publication bias with Egger’s test for continuous variables and Harbord’s test for dichotomous variables. Funnel plots were generated for analyses involving more than ten studies, and were visually inspected for asymmetrical distribution of data points across the vertical treatment effect axis.^32,33^

### Role of the funding source

There was no funding source for this study.

## Results

**Figure 1:**
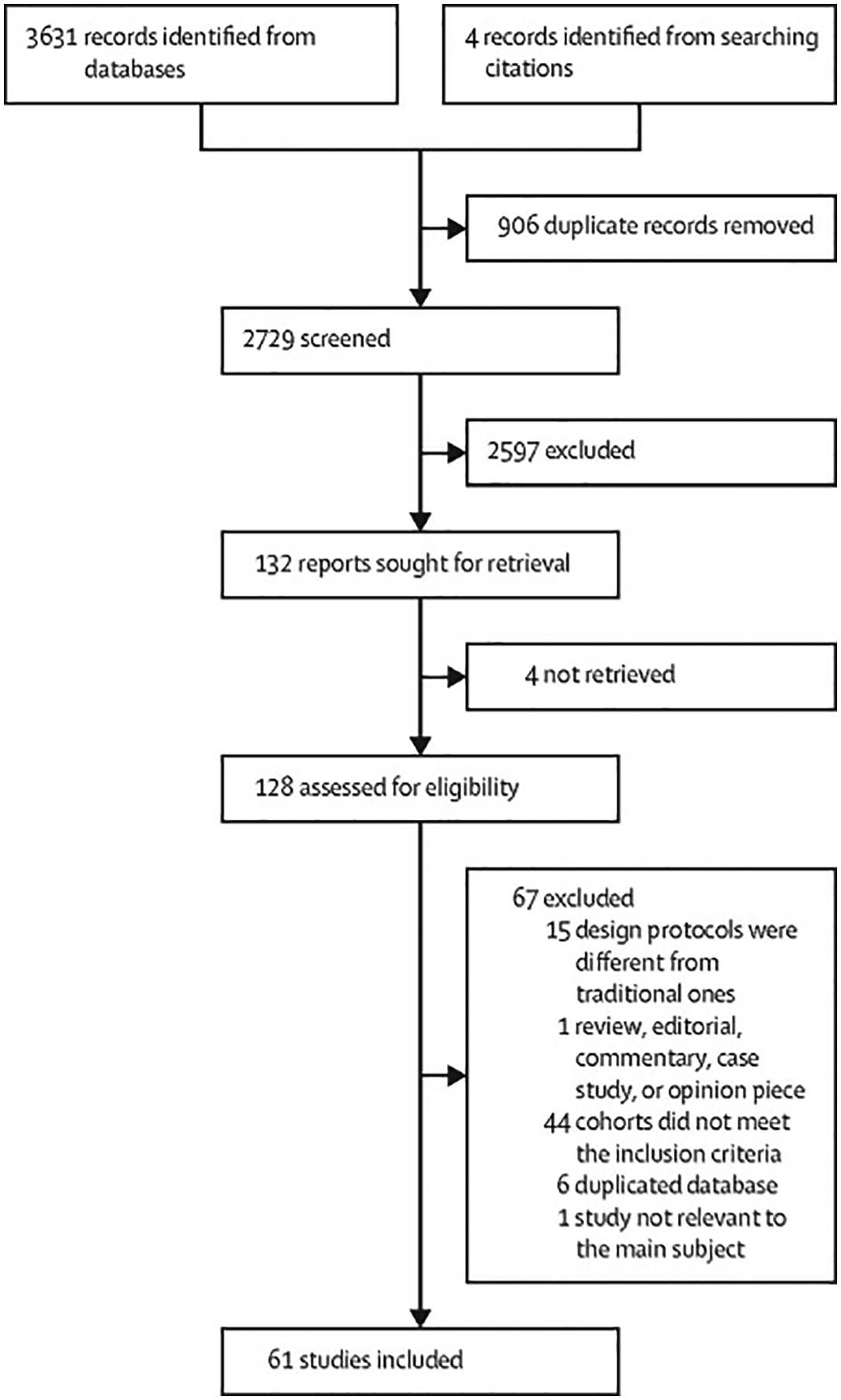
Study selection

The initial search from MEDLINE and Embase yielded 3631 articles. After screening, 61 studies (done between January, 1980, and May, 2021) were included in the meta-analysis (figure 1). The included studies were done in Argentina, Austria, Belgium, Canada, mainland China, France, Germany, India, Italy, Japan, Latin America, the Netherlands, Singapore, South Korea, Slovakia, Sweden, Taiwan, the UK, and the USA. Additionally, one study was a European multicentre study, and another was a multicentre study involving centres from Germany, Italy, Japan, and South Korea. A total of 94 636 patients were included: 15 377 patients with NAFLD-related hepatocellular carcinoma and 79 259 patients with hepatocellular carcinoma due to other causes. The median age of included patients was 67·8 years (IQR 60·2– 74·5). The quality of included studies, based on the JBI Checklist, was generally moderate to good (appendix pp 2–9).

**Figure 2:**
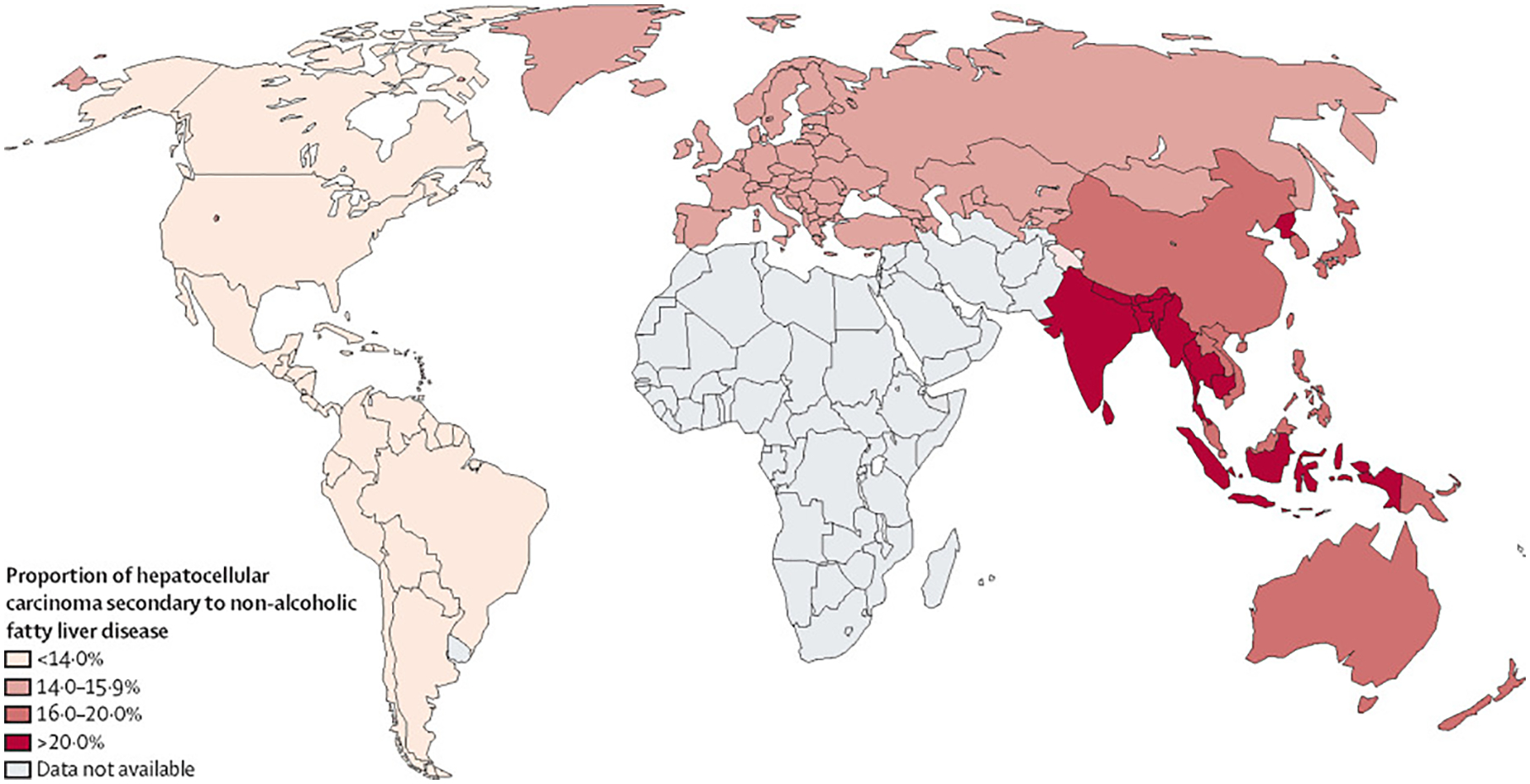
Proportion of hepatocellular carcinoma secondary to non-alcoholic fatty liver disease worldwide, by WHO region

The overall proportion of patients with hepatocellular carcinoma secondary to NAFLD was 15·1% (95% CI 11·9–18·9). The proportion of patients with NAFLD-related hepatocellular carcinoma was highest in the South-East Asia region, followed by the Western Pacific region, European region, and the region of the Americas (figure 2; appendix p 10–11). There was an increase in the global proportion of hepatocellular carcinoma secondary to NAFLD over time periods (9·77%, 95% CI 7·33–12·92 for before 2000 *vs* 16·97%, 12·17–23·16 for 2010 and beyond; p=0·045; appendix pp 10–11). NAFLD-related hepatocellular carcinoma did not differ between clinical cohort studies versus administrative database studies (post-hoc analysis; appendix p 11).

Patients with NAFLD-related hepatocellular carcinoma were older (mean difference 5·62 years; 95% CI 4·63–6·61; p<0·0001), and had higher BMI (mean difference 2·99 kg/m^2^, 2·20–3·78; p<0·0001) compared with patients with hepatocellular carcinoma due to other causes (table 1). There was no significant difference in terms of sex between the two groups. Patients with NAFLD-related hepatocellular carcinoma were also more likely to present with metabolic complications, including diabetes (OR 4·31, 95% CI 3·19–5·80; p<0·0001), hypertension (2·84, 2·09–3·86; p<0·0001), and hyperlipidaemia (3·43, 2·39–4·95; p<0·0001) than were patients with hepatocellular carcinoma due to other causes (table 1). Patients with NAFLD-related hepatocellular carcinoma were also more likely to have cardiovascular disease at presentation than were patients with hepatocellular carcinoma due to other causes (2·23, 1·43–3·48; p=0·0055). Additional subgroup analyses comparing the clinical characteristics of NAFLD-related hepatocellular carcinoma versus hepatocellular carcinoma secondary to HBV, HCV, and alcohol consumption are summarised in the appendix (pp 12, 16).

**Figure 3:**
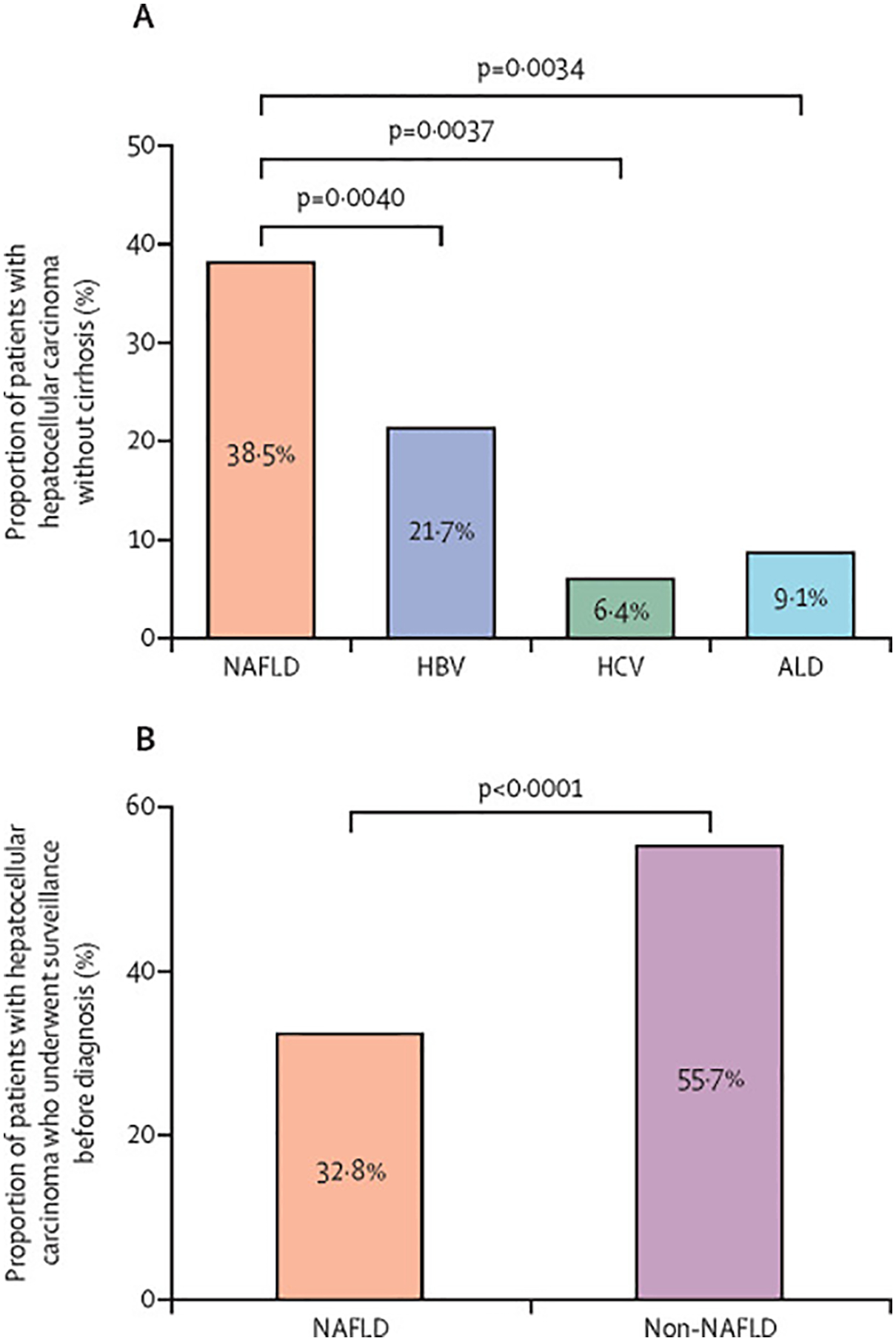
Proportion of hepatocellular carcinoma Patients without cirrhosis (A) and patients who underwent surveillance before hepatocellular carcinoma diagnosis (B). HBV=hepatitis B virus. HCV=hepatitis C virus. NAFLD=non-alcoholic fatty liver disease. ALD=alcohol-associated liver disease.

A higher proportion of patients with NAFLD-related hepatocellular carcinoma did not have cirrhosis (38·5%, 95% CI 27·9–50·2) compared with patients with hepatocellular carcinoma secondary to other causes (14·6%, 8·7–23·4; p<0·0001). The proportion of patients without cirrhosis and with hepatocellular carcinoma related to HBV was 21·7% (14·0–31·9), to HCV was 6·4% (2·1–17·6), and to alcohol was 9·1% (4·0–19·0; figure 3A). The odds of not having cirrhosis in NAFLD-related hepatocellular carcinoma versus hepatocellular carcinoma of other causes was increased (OR 3·71, 95% CI 2·46–5·59; p<0·0001). Patients with NAFLD-related hepatocellular carcinoma were more likely to be non-cirrhotic than were patients with hepatocellular carcinoma secondary to HBV (3·28, 1·68–6·43; p=0·0040), HCV (6·41, 2·30–17·91; p=0·0037), and alcohol (3·13, 1·66–5·90; p=0·0034).

Patients with NAFLD-related hepatocellular carcinoma had a larger tumour diameter (mean difference 0·67 cm, 95% CI 0·35–0·98; p=0·0087) and greater odds of having uninodular lesions (OR 1·36, 95% CI 1·19–1·56; p=0·0003) than had patients with hepatocellular carcinoma due to other causes (table 1). However, the odds of patients with BCLC stage 0 or A, BCLC stage B, BCLC stage C or D, TNM stage 1 or 2, TNM stage 3 or 4, or ECOG of 2 or worse at presentation did not differ (table 1); alpha fetoprotein concentration was also not significantly different between groups. Subgroup analyses comparing tumour characteristics of NAFLD-related hepatocellular carcinoma versus hepatocellular carcinoma secondary to HBV, HCV, and alcohol are summarised in the appendix (pp 13–15).

Only 32·8% (95% CI 12·0–63·7; four studies, 393 patients) of patients with NAFLD-related hepatocellular carcinoma had surveillance for hepatocellular carcinoma before cancer diagnosis versus 55·7% (24·0–83·3; eight studies, 3583 patients) of patients with hepatocellular carcinoma secondary to other causes (OR 0·36, 95% CI 0·28–0·48; p<0·0001; four studies, 2826 patients; figure 3B).

Overall, 65·0% (95% CI 30·4–88·8) of patients with NAFLD-related hepatocellular carcinoma were assigned to curative therapy and 42·1% (24·8–61·6) of patients to palliative therapy. Best supportive care could not be assessed due to insufficient studies. A prespecified subgroup analysis identified that the proportion of patients with NAFLD-related hepatocellular carcinoma who received liver transplant was 3·9% (1·4–12·3), who received resection was 33·6% (11·1–67·1), and who received ablation was 12·0% (6·9–20·1). The odds of receiving curative therapy did not differ between NAFLD-related versus non-NAFLD-related hepatocellular carcinoma (OR 1·05, 95% CI 0·52–2·09; p=0·90; table 2). However, patients with NAFLD-related hepatocellular carcinoma were less likely to undergo liver transplant (p=0·017) but more likely to undergo liver resection (p=0·0090) than patients with hepatocellular carcinoma due to other causes (table 2). Patients with NAFLD-related hepatocellular carcinoma had similar odds to patients with hepatocellular carcinoma due to other causes (p=0·18) of receiving ablation (table 2). The odds of receiving palliative therapy (1·01, 0·72–1·42; p=0·76) or best supportive care (1·46, 0·77–2·77; p=0·20) did not differ between NAFLD-related versus non-NAFLD-related hepatocellular carcinoma (table 2). Subgroup analysis for treatment allocation by cause of liver disease could not be done due to a lack of data in available studies.

The factors associated with receiving curative treatment among patients with NAFLD-related hepatocellular carcinoma are summarised in the appendix (p 17).

Overall survival did not differ between NAFLD-related versus non-NAFLD-related hepatocellular carcinoma (HR 1·05, 95% CI 0·92–1·20; p=0·43; table 2). Subgroup analyses also did not differ for overall survival between NAFLD-related hepatocellular carcinoma and hepatocellular carcinoma secondary to HBV, HCV, or alcohol (appendix pp 19–20). However, among patients with cirrhosis (sensitivity analysis), NAFLD-related hepatocellular carcinoma was associated with increased mortality (p=0·0041) compared with hepatocellular carcinoma due to other causes (1·74, 1·21–2·67; p=0·0041). A sensitivity analysis of patients who underwent curative therapy established that NAFLD-related hepatocellular carcinoma was associated with longer overall survival than HCV-related hepatocellular carcinoma for patients receiving curative treatment (p=0·041; appendix p 20), but not when compared with HBV-related hepatocellular carcinoma (p=0·78; appendix p 20) or all other causes combined (p=0·94; table 2). For the sensitivity analysis for type of curative treatment, overall survival did not differ between NAFLD-related hepatocellular carcinoma compared with other causes in patients receiving liver transplant (p=0·13) or resection (p=0·58; table 2). Meta-regression of study level data did not reveal any study-level factors to be associated with overall survival among patients with NAFLD-related hepatocellular carcinoma (appendix p 18).

NAFLD-related hepatocellular carcinoma had improved disease-free survival compared with non-NAFLD-related hepatocellular carcinoma (HR 0·79, 95% CI 0·63–0·99; p=0·044; table 2), although subgroup analysis by specific causes (HBV, HCV, and alcohol) did not differ (appendix p 20). Disease-free survival among patients with cirrhosis was not analysed due to insufficient data. In a sensitivity analysis, NAFLD-related hepatocellular carcinoma was associated with improved disease-free survival among patients who received curative therapy (p=0·0011). In sensitivity analysis for specific types of curative treatment, NALFD-related hepatocellular carcinoma was also associated with improved disease-free survival in patients who underwent liver resection (p=0·0043; table 2), but not for liver transplantation (p=0·12). Meta-regression of study-level data revealed that only increased alpha fetoprotein was associated with reduced disease-free survival, all other assessed risk factors were not associated with disease-free survival (p=0·029; appendix p 18).

There was substantial heterogeneity in most analyses (*I*^2^>75%). Additionally, there was publication bias in the analysis of baseline characteristics between NAFLD-related and non-NAFLD-related hepatocellular carcinoma, notably in the analysis of BMI (p=0·032), diabetes (p=0·0042), and hypertension (p=0·020), but not age (p=0·50; appendix pp 21–23) or male gender (p=0·50). No publication bias was noted in the analysis for cirrhosis, tumour diameter, uninodular cancer, BCLC stage, treatment allocation, overall survival, and disease-free survival between NAFLD-related and non-NAFLD-related hepatocellular carcinoma (appendix pp 24–28).

## Discussion

In this large systematic review and meta-analysis of 61 studies and 94 636 individuals, globally, about 15% of hepatocellular carcinoma were secondary to NAFLD. The highest proportion of NAFLD-related hepatocellular carcinoma occurred in the South-East Asia region, and the lowest in the region of the Americas. The global proportion of hepatocellular carcinoma secondary to NAFLD increased over time. Patients with NAFLD-related hepatocellular carcinoma were older, had higher BMI, and had more metabolic comorbidities than patients with hepatocellular carcinoma due to other causes. Nearly 40% of patients with NAFLD-related hepatocellular carcinoma did not have cirrhosis, compared with about 15% of patients with hepatocellular carcinoma due to other causes. In addition, only 33% of patients with NAFLD-related hepatocellular carcinoma underwent surveillance before hepatocellular carcinoma diagnosis, versus 56% of patients with hepatocellular carcinoma due to other causes. These findings have important implications. Because of the absence of cirrhosis, nearly 40% of patients with NAFLD-related hepatocellular carcinoma did not have routine indication for hepatocellular carcinoma surveillance before hepatocellular carcinoma diagnosis based on current practice guidelines.^34,35^ This probably contributed to the dismal rate of hepatocellular carcinoma surveillance among patients with NAFLD-related hepatocellular carcinoma, in addition to poor disease awareness.^36,37^ Several studies have shown that NAFLD is severely underdiagnosed in clinical practice and associated with high mortality.^38,39^ In addition, patients with NAFLD are often overweight, which reduces the sensitivity of ultrasound to detect early hepatocellular carcinoma, and alternative methods for hepatocellular carcinoma screening might be required for patients with poor liver visualisation on ultrasound.^3,40,41^ Better strategies are required to identify non-cirrhotic patients with NAFLD at high risk of hepatocellular carcinoma who require surveillance.^42^

Patients with NAFLD-related hepatocellular carcinoma had larger tumours at diagnosis than patients with hepatocellular carcinoma due to other causes, but were more likely to have uninodular lesions. This occurrence might be related to the higher proportion of patients with NAFLD-related hepatocellular carcinoma without cirrhosis, as several studies have reported a larger tumour size in livers without cirrhosis and a higher proportion of uninodular lesions than for livers with cirrhosis.^43–45^ Larger tumour size might be related to the lower resistance to expansive tumour growth in non-cirrhotic livers, but more data are required to validate this hypothesis.^43,44^ The odds of BCLC stage 0 or A at diagnosis were similar between NAFLD-related hepatocellular carcinoma versus hepatocellular carcinoma of other causes despite the larger tumour size, which might be related to the higher odds of uninodular disease in NAFLD-related hepatocellular carcinoma. However, the similar odds of BCLC stage 0 or A between NAFLD-related and non-NAFLD-related hepatocellular carcinoma should be interpreted with caution. There were insufficient data to establish the effect of lower surveillance rates in NAFLD-related hepatocellular carcinoma on the proportion of BCLC stage 0 or A at presentation as only four of 11 cohorts that provided data for BCLC stage underwent hepatocellular carcinoma surveillance. More data are required to establish the effect of lower hepatocellular carcinoma surveillance rates in NAFLD-related hepatocellular carcinoma on tumour burden at diagnosis. Compared with hepatocellular carcinoma due to other causes, patients with NAFLD-related hepatocellular carcinoma were less likely to receive liver transplantation and more likely to receive liver resection, but had a similar likelihood of receiving ablation. The higher odds of receiving resection and lower odds of receiving liver transplantation might be related to the higher proportion of patients with NAFLD-related hepatocellular carcinoma without cirrhosis. In addition, the older age, higher BMI, and higher proportion of patients with cardiovascular disease probably contributed to the lower proportion of patients with NAFLD receiving liver transplant. Overall, patients with NAFLD-related hepatocellular carcinoma had similar odds of receiving curative therapy versus patients with hepatocellular carcinoma due to other causes. Meta-regression showed that older age and the presence of cirrhosis were associated with a lower likelihood of receiving curative therapy. However, among patients with cirrhosis, NAFLD-related hepatocellular carcinoma was associated with increased mortality versus hepatocellular carcinoma due to other causes.

Patients with NAFLD-related hepatocellular carcinoma had similar overall survival versus patients with hepatocellular carcinoma due to other causes, although overall survival was longer among patients with NAFLD-related hepatocellular carcinoma who had undergone curative therapy than among those with HCV-related hepatocellular carcinoma who had undergone curative therapy. However, most of the included studies were conducted before the widespread availability of direct-acting antivirals for HCV and the comparative survival outcomes between NAFLD-related hepatocellular carcinoma and HCV-related hepatocellular carcinoma are likely to change as more patients with HCV are treated with direct-acting antivirals. Patients with NAFLD-related hepatocellular carcinoma had improved disease-free survival versus patients with hepatocellular carcinoma due to other causes in the overall analysis. This outcome remained true in subgroup analysis of patients who had undergone curative therapy, and those patients who underwent liver resection. However, in patients who received liver transplantation, disease-free survival was similar between NAFLD-related and non-NAFLD-related hepatocellular carcinoma. This outcome might be related to the higher proportion of patients with NAFLD-related hepatocellular carcinoma without cirrhosis, as cirrhosis increases the risk of hepatocellular carcinoma recurrence after resection.^46,47^

Several studies have reported improved outcomes in NAFLD-related compared with non-NAFLD-related hepatocellular carcinoma,^48,49^ although there have also been reports of more advanced disease at presentation and poorer outcomes for NAFLD-related hepatocellular carcinoma.^14,50^ These conflicting results could have arisen from previous studies being specific to a country, region, or treatment, hence limiting their generalisability. The current study provides a comprehensive global overview of the clinical presentation, treatment allocation, and outcomes of NAFLD-related hepatocellular carcinoma. In addition, we did multiple subgroup analyses to account for causes, treatment type, region, time period, and study setting to inform practice, with most of the included studies of at least moderate-to-high quality.

To our knowledge, the current study provides the most comprehensive analysis of the existing literature regarding clinical characteristics and outcomes of NAFLD-related hepatocellular carcinoma to date. However, our study is not without limitations, some of which are inherent to the nature of meta-analyses.^51,52^ There was substantial heterogeneity in certain comparisons, which could have been arisen from the large sample size involved in pooled analysis. We attempted to account for possible sources of heterogeneity by conducting subgroup analyses and meta-regression where appropriate. There were also insufficient studies that provided comparative data for the tumour characteristics of patients without cirrhosis. In addition, there were relatively few studies from the South-East Asia region and South America, and the number of included patients were modest, therefore, the analyses for the proportion of hepatocellular carcinoma secondary to NAFLD from these regions require cautious interpretation and more data are required. However, the high proportion of hepatocellular carcinoma secondary to NAFLD in the South-East Asia region is consistent with a large meta-analysis reporting an NAFLD prevalence of 42·04% in South-East Asia, which probably contributes to the high proportion in the region.^5,53^ However, data regarding the contribution of genetic variants to the risk of NAFLD-related hepatocellular carcinoma from the South-East Asia region are lacking. There was a paucity of data from the African region and the Eastern Mediterranean region. In addition, the various causes of liver disease are not well distributed geographically, therefore, it is possible that pooling data from different regions into a single estimate might result in subtle bias. However, we attempted to mitigate this effect by performing extensive subgroup analyses stratified by both cause and region where possible to provide granular data. Finally, as the included studies used a combination of clinical, radiological, and histological assessments to diagnose cirrhosis, the proportion of patients without cirrhosis might have been overestimated.

This meta-analysis provides a comprehensive global overview of the clinical presentation, surveillance rates, treatment allocation, and outcomes of NAFLD-related hepatocellular carcinoma. This study provides high-level evidence that a substantially higher proportion of patients with NAFLD-related hepatocellular carcinoma do not have cirrhosis and have lower surveillance rates than have patients with hepatocellular carcinoma due to other causes. The proportion of hepatocellular carcinoma secondary to NAFLD is rising globally, and urgent measures are required to tackle the metabolic risk factors associated with NAFLD-related hepatocellular carcinoma. Further studies are required to improve hepatocellular carcinoma surveillance strategies for patients with NAFLD who are at high-risk of hepatocellular carcinoma without cirrhosis.

## Supplementary Material

Supplementary Appendix

## Figures and Tables

**Table 1:** Clinical and tumour characteristics of NAFLD-related hepatocellular carcinoma versus hepatocellular carcinoma due to other causes

	Studies, n	Patients, n	Effect size (95% CI)	p value	Cochran’s Q	*I* ^2^
**Patient characteristics**						
Age, years	35	73 028	MD 5·62 (4·63 to 6·61)	<0·0001	<0·0001	94·6%
Body-mass index, kg/m^2^	21	45 560	MD 2·99 (2·20 to 3·78)	<0·0001	<0·0001	96·1%
Men	35	72 021	OR 0·71 (0·39 to 1·29)	0·25	<0·0001	98·2%
Women	35	72 021	OR 1·41 (0·77 to 2·57)	0·25	<0·0001	98·1%
Diabetes	26	43 406	OR 4·31 (3·19 to 5·80)	<0·0001	<0·0001	91·8%
Hypertension	18	6147	OR 2·84 (2·09 to 3·86)	<0·0001	<0·0001	90·3%
Hyperlipidaemia	8	4240	OR 3·43 (2·39 to 4·95)	<0·0001	0·083	44·4%
Cardiovascular disease	6	21 223	OR 2·23 (1·43 to 3·48)	0·0055	<0·0001	94·2%
Cirrhosis	27	21 791	OR 0·27 (0·18 to 0·41)	<0·0001	<0·0001	89·2%
No cirrhosis	27	21 791	OR 3·71 (2·46 to 5·59)	<0·0001	<0·0001	89·1%
**Tumour characteristics**						
Tumour diameter, cm	18	11 639	MD 0·67 (0·35 to 0·98)	0·0087	<0·0001	79·0%
Uninodular hepatocellular carcinoma	13	9345	OR 1·36 (1·19 to 1·56)	0·0003	0·51	0
Alpha fetoprotein	17	18 637	MD –0·04 (–0·16 to 0·07)	0·47	<0·0001	75·6%
BCLC O or A	9	7506	OR 1·14 (0·72 to 1·80)	0·53	0·0009	69·2%
BCLC B	11	8925	OR 1·27 (0·86 to 1·90)	0·21	<0·0001	79·9%
BCLC C or D	11	8925	OR 1·28 (0·70 to 2·36)	0·38	<0·0001	91·9%
TNM 1 or 2	3	250	OR 1·82 (0·30 to 11·16)	0·29	0·30	16·0%
TNM 3 or 4	3	250	OR 0·81 (0·23 to 2·87)	0·54	0·54	0
ECOG performance status of 2 or worse	3	3142	OR 1·17 (0·71 to 1·92)	0·32	0·52	0

NAFLD=non-alcoholic fatty liver disease. MD=mean difference. OR=odds ratio. BCLC=Barcelona Clinic Liver Cancer. ECOG=Eastern Cooperative Oncology Group.

**Table 2:** Treatment allocation and survival outcomes between NAFLD-related hepatocellular carcinoma versus hepatocellular carcinoma due to other causes

	Studies, n	Events, n	Patients, n	Effect size (95% CI)	p value	Cochran’s Q	*I* ^2^
**Overall treatment allocation**							
Curative therapy*	18	23 601	30 082	OR 1·05 (0·52–2·09)	0·90	<0·0001	88·8%
Palliative therapy†	13	3026	8100	OR 1·01 (0·72–1·42)	0·76	<0·0001	95·2%
Best supportive care	7	1948	4799	OR 1·46 (0·77–2·77)	0·20	<0·0001	80·4%
**Method of curative treatment**							
Liver transplantation	8	457	5177	OR 0·49 (0·29–0·84)	0·017	0·028	55·3%
Liver resection	12	1229	7171	OR 1·59 (1·16–2·18)	0·0090	0·026	52·4%
Ablation	10	1188	6495	OR 0·88 (0·71–1·08)	0·18	0·57	0
**Survival outcomes**							
Overall survival							
Overall	28	··	39 585	HR 1·05 (0·92–1·20)	0·43	<0·0001	99·5%
All curative therapies*‡	13	··	7168	HR 1·00 (0·85–1·16)	0·94	0·073	40·4%
Liver transplantation	3	··	1811	HR 0·74 (0·50–1·10)	0·13	0·61	0
Liver resection	6	··	3325	HR 1·13 (0·72–1·79)	0·58	<0·0001	75·0%
Disease-free survival							
Overall	16	··	5922	HR 0·79 (0·63–0·99)	0·044	0·018	47·3%
All curative therapies*‡	10	··	4984	HR 0·76 (0·64–0·89)	0·0011	0·28	17·5%
Liver transplantation	3	··	1445	HR 0·56 (0·27–1·15)	0·12	0·12	53·0%
Liver resection	6	··	3325	HR 0·82 (0·71–0·95)	0·0043	0·90	0

NAFLD=non-alcoholic fatty liver disease. OR=odds ratio. HR=hazard ratio.

* Includes liver transplantation, resection, and ablation.

† Includes transarterial chemoembolisation, radioembolisation, radiotherapy, and systemic therapy.

‡ The remaining treatment methods for curative therapies (ablation) had insufficient studies for pooled analysis to be done.

## Data Availability

All articles in this Article are available from MEDLINE and Embase.

## Research in context

### Evidence before this study

Data regarding the clinical presentation and outcomes for non-alcoholic fatty liver disease (NAFLD)-related hepatocellular carcinoma versus hepatocellular carcinoma due to other causes are conflicting. We searched PubMed for systematic reviews published from database inception until Jan 17, 2022, using search terms “non-alcoholic fatty liver disease” and “hepatocellular carcinoma”, but did not identify any previous comprehensive meta-analysis evaluating the overall clinical presentation, surveillance rates, and survival outcomes between NAFLD-related and non-NAFLD-related hepatocellular carcinoma.

### Added value of this study

Unlike previous meta-analyses on this topic that were specific to a treatment, country, or region, this study provides a comprehensive global overview of the proportion of hepatocellular carcinoma secondary to NAFLD, clinical presentation, treatment allocation, and outcomes of NAFLD-related hepatocellular carcinoma. This study determined that the global proportion of hepatocellular carcinoma secondary to NAFLD is rising. In addition, this meta-analysis provides high-level evidence that a higher proportion of patients with NAFLD-related hepatocellular carcinoma do not have cirrhosis and these patients have substantially lower surveillance rates than those with hepatocellular carcinoma due to other causes.

### Implications of all the available evidence

Many patients with NAFLD-related hepatocellular carcinoma do not have cirrhosis and lack a routine indication for hepatocellular carcinoma surveillance based on current practice guidelines. This contributes to few patients with NAFLD-related hepatocellular carcinoma receiving surveillance before diagnosis. Further studies are required to improve hepatocellular carcinoma surveillance strategies for patients with NAFLD and without cirrhosis who are high risk. Urgent measures are required to tackle the underlying metabolic risk factors and reduce the growing burden of NAFLD-related hepatocellular carcinoma.
