# Supplementary material for: Clinical characteristics, surveillance, treatment allocation, and outcomes of non-alcoholic fatty liver disease-related hepatocellular carcinoma: a systematic review and meta-analysis
Source: Lancet Oncol. Author manuscript; Available in PMC 2022 Dec 2. (PMC9718369; doi:10.1016/S1470-2045(22)00078-X)
Supplement: Supplementary Appendix [file NIHMS1852064-supplement-Supplementary_Appendix.pdf]

THE LANCET
Oncology

Supplementary appendix

This appendix formed part of the original submission and has been peer reviewed. We post it as supplied by the authors.

Supplement to: Tan DJH, Ng CH, Lin SY, et al. Clinical characteristics, surveillance, treatment allocation, and outcomes of non-alcoholic fatty liver disease-related hepatocellular carcinoma: a systematic review and meta-analysis. *Lancet Oncol* 2022; published online March 4. https://doi.org/10.1016/S1470-2045(22)00078-X.

Supplementary Material 1: Search strategy

1: ('hcc' or 'hepatocellular carcinoma' or ‘hepatic cell carcinoma’ or ‘liver neoplasm’ or ‘hepatoma’ or ‘liver cell carcinoma’ or ‘primary liver carcinoma’):ti,ab or ‘liver cell carcinoma’/exp

2: ‘nonalcoholic fatty liver/exp or ('nafld’ or ‘nash’ or ‘fatty liver’ or ‘steatohep*’ or ((‘nonalcoholic’ or ‘non alcoholic’ or non-alcoholic’) and (‘fatty liver’ or ‘steatohep*’))):ti,ab

3: (outcome* or 'natural history' or’ prognos*’ or ‘progression’ or ‘disease’ or ‘course’ or ‘evolution’):ti,ab

4: ‘controlled study’/exp or ‘randomized controlled trial/exp or ((‘control$’ or ‘randomized’) near/2 (‘study’ or ‘studies’ or ‘trial’ or ‘trials’)):ti,ab or (‘prospective’ and (‘study’ or ‘studies’ or ‘survey’ or ‘surveys’ or ‘analysis’ or ‘analyses’ or ‘trial*’)):ti,ab

5: 1 and 2 and 3 and 4

Supplementary Material 2: Summary of included articles

Author, year	Country/ region	Study Duration	Non-NAFLD etiologies	Total population (HCC)	Sample Size (NAFLD)	Sample Size (Non-NAFLD)	Sample Size (HBV)	Sample Size (HCV)	Sample Size (ALD)	Age (mean years,sd)	Gender Male (%)	Quality Assessment
Lim et al, 2021[1] *	Singapore	2008-2018	HBV, HCV, ALD, AIH, PBC	1186	321	865	553	124	179	-	-	7
Lin et al, 2021[2]	Taiwan	2011-2019	HBV	179	23	156	156	-	-	70.0 (14.2)	72.6	8
Pinyopornpanish et al, 2021[3]	USA	2000-2015	HBV, HCV, ALD, AIH, PBC	2237	587	1650	129	873	293	-	-	8
Safcak et al, 2021[4]	Slovakia	2010-2016	HBV, HCV, ALD	368	54	314	-	-	-	69.8 (8.8)	66.6	7
Jung et al, 2021[5]	South Korea	2005-2015	HBV	232	32	200	200	-	-	61.0 (11.0)	73.7	8
Hiraoka et al, 2021[6]	Japan	2018-2021	HBV, HCV, ALD	530	103	427	-	-	-	74.7 (8.3)	78.8	8
Kumar et al, 2020[7]	Singapore	2000-2013	ALD	99	54	45	-	-	45	72.0 (9.0)	78.3	8
Paik et al, 2020[8]	USA	2007-2017	HBV, HCV, ALD	11765	4291	7474	727	6099	648	70.3 (12.5)	78.3	7
Ahn et al, 2020[9]	South Korea	2000-2016	HBV, ALD	622	56	566	393	-	173	68.0 (10.9)	82.6	7
Hanumanthappa et al, 2020[10]	Canada	2011-2015	HBV, HCV	195	12	183	18	37	-	-	-	8
Wang et al, 2020[11]	USA	2010-2019	HBV, HCV, ALD, AIH	6346	1795	4551	355	1563	2399	-	-	7
Liew et al, 2019[12] *	Singapore	1980-2015	HBV	1079	163	916	916	-	-	67.6 (10.8)	81.7	8
Tateishi et al, 2019[13]	Japan	2011-2015	ALD, AIH, PBC	1106	315	791	-	-	675	-	-	7
Bengtsson et al, 2019[14]	Sweden	2004-2017	HBV, HCV, ALD, AIH, PBC	1562	225	1337	-	-	-	71.3 (7.5)	77.1	8
Patkar et al, 2019[15]	India	2009-2017	HBV, HCV, ALD	105	40	65	42	10	13	-	-	8
Hester et al, 2019[16]	USA	2008-2016	HBV, HCV, ALD	1051	92	959	87	719	153	67.9 (10.8)	79.5	8
Pinero et al, 2018[17]	Latin America	2005-2012	HBV, HCV, ALD, AIH	435	25	410	110	159	73	60.0 (4.0)	81.8	7

Pinero et al, 2018[18]	Argentina	2009-2016	HBV, HCV, ALD, AIH	708	81	627	38	262	147	63.0 (8.0)	75.8	7
Ioannou et al, 2018[19]	USA	2001-2017	HCV, ALD	9601	608	8993	-	7605	1388	66.4 (8.1)	6.23	8
Kim et al, 2018[20]	USA	1998-2015	HBV, HCV, ALD	1884	95	1789	465	1194	130	-	-	8
Sadler et al, 2018[21]	USA	2004-2014	HBV, HCV, ALD	929	60	869	216	522	90	63.7 (5.4)	80.2	7
Yoon et al, 2018[22]	South Korea	2005-2015	HBV, HCV, ALD	1260	63	1197	869	137	191	68.0 (13.0)	80.3	8
Marot et al, 2017[23]	Belgium	1995-2014	HCV, ALD	85	12	73	-	35	38	-	-	8
Kimura et al, 2017[24]	Japan	2996-2012	ALD	61	30	31	-	-	-	68.8 (6.1)	96.7	7
Pais et al, 2016[25]	Italy	1995-2014	HBV, HCV, ALD	323	39	284	-	-	-	70.0 (9.0)	79.9	8
Than et al, 2017[26]	United Kingdom	2000-2014	HCV	487	212	275	-	275	-	69.6 (8.7)	80.1	7
Piscaglia et al, 2016[27]	Italy	2010-2012	HCV	756	145	611	-	-	-	67.8 (9.0)	64.7	8
Meer et al, 2016[28]	Netherlands	2005-2012	HBV, HCV, ALD	976	181	795	249	197	349	-	-	8
Lopes et al. 2016[29]	Brazil	2000-2014	HBV, HCV, ALD	66	8	58	11	34	13	-	-	7
Mittal et al, 2015[30] ^	USA	2005-2010	HCV, ALD	1419	120	1299	-	1013	286	74.7, -	99.8	7
Younossi et al, 2015[31]	USA	2004-2009	HBV, HCV, ALD	4725	701	4024	471	2736	817	73.2 (8.1)	71.6	9
Beste et al, 2015[32] ^	USA	1999-2014	HBV, HCV, ALD, AIH	7313	1029	6284	176	5225	873	70.5 (10.2)	16.3	7
Tateishi et al, 2015[33]	Japan	1991-2010	ALD, AIH	2180	596	1584	-	-	1423	71.7 (8.2)	78.8	9
Dyson et al, 2014[34]	United Kingdom	2000-2010	HBV, HCV, ALD, AIH	416	136	280	29	65	178	71.0, -	84.1	8
Paranaguá-Vezozzo et al, 2014[35]	Brazil	1998-2008	HBV, HCV, ALD	69	1	68	16	47	5	-	-	8
Weinmann et al, 2014[36]	Germany	2004-2009	HBV, HCV, AIH	283	28	255	79	174	-	-	-	8

Teixeira et al, 2012[37]	Brazil	2001-2009	HBV, HCV	9	5	4	2	2	-	-	-	7
Arase et al, 2012[38]	Japan	1994-2007	HCV	277	10	267	-	267	-	-	-	9
Hernandez-Alejandrio et al, 2012[39]	Canada	2000-2011	HCV	81	17	64	-	64	-	58.6 (4.2)	94.0	7
Reddy et al, 2012[40]	USA	2000-2012	HCV, ALD	214	54	162	-	-	-	64.0 (9.9)	75.7	9
Hucke et al, 2011[41]	Austria	1991-2009	HBV, HCV, ALD	387	23	364	30	127	207	-	-	8
Ascha et al, 2010[42]	USA	2003-2007	HCV	89	25	64	-	64	-	-	-	9
Tokushige et al, 2010[43]	Japan	1990-2007	HCV	90	34	56	-	56	-	70.1 (7.6)	60.0	8
Sanyal et al, 2006[44]	USA	1992-2004	HCV	6	3	3	-	3	-	-	-	9
Hashimoto et al, 2004[45]	Japan	1989-2003	ALD	58	8	50	-	-	50	67.3 (19.7)	78.5	8
Shahid et al, 2009[46]	USA	1997-2008	HBV, HCV, ALD, AIH	447	27	430	52	138	99	63.1 (6.4)	-	7
Amarapurkar et al, 2013[47]	India	2010-2011	HBV, HCV	46	6	40	26	14	-	-	-	9
Jain et al, 2012[48]	India	2005-2010	ALD	13	8	5	-	-	5	59.6 (3.0)	100	8
Judith et al, 2010[49]	Germany	2007-2008	HBV, HCV, ALD	119	36	83	29	35	19	68.6 (8.4)	84.0	7
Schutte et al, 2014[50]	Germany	1994-2003	Viral, ALD	440	43	397	-	-	299	-	-	8
Koh et al, 2019[51] *	Singapore	2000-2015	Non-NAFLD	996	152	844	-	-	-	68.0 (8.2)	77.7	9
Yang et al, 2019[52]	China	2003-2014	HBV	1483	96	1387	1387	-	-	57.3 (12.5)	88.5	8
Wakai et al, 2011[53]	Japan	1990-2007	HBV, HCV	225	17	208	61	147	-	-	-	8
Morisco et al, 2018[54]	Italy	1987-2014	Viral, ALD	839	190	645	-	-	159	-	-	8
Gawrieh et al, 2019[55]	USA	2000-2014	HBV, HCV, ALD, AIH	3076	767	2309	315	1263	655	-	-	7

D'Silva et al, 2021[56]	South Korea	2004-2018	HBV	575	36	539	36	-	-	-	-	7
Rimini et al, 2021[57]	Multicentre (Japan, Korea, Germany and Italy)	2010-2021	HCV, HBV	1232	236	996	268	453	-	72.0 (8.25)	78.0	7
Halder et al, 2019[58]	Europe	2002-2016	Non-NAFLD	20195	1073	19122	-	-	-	61.0 (5.9)	83.7	7
Kalaitzakis et al, 2011[59]	Europe	1994-2005	HBV, HCV, ALD, AIH, Viral, PBC/PSC	114	20	94	7	20	34	-	-	7
Nilsson et al, 2019[60]	Europe	2011-2010	HCV, ALD, AIH, PBC/PSC	202	32	170	-	58	57	-	-	7
Tohra et al, 2021[61]	India	2007-2015	HBV, HCV, ALD, AIH, PBC/PSC	785	138	647	158	133	141	60 (7.97)	87.2	7

* Lim et al. 2021, Liew et al. 2019, and Koh et al. 2019 included patients from the same institution (Singapore General Hospital). To avoid overlapping of cohorts, data from Liew et al. were only included in the analysis of the proportion of HCC patients that underwent surveillance before diagnosis, and data from Koh et al. were only included in the analysis of curative treatment allocation.

^ Mittal et al. 2015 and Beste et al. 2015 utilized data from the United States Veterans Administration database. To avoid overlapping of cohorts, data from Mittal et al. were only included in the analysis of the proportion of HCC patients that underwent surveillance before diagnosis.

Abbreviations: HBV, hepatitis B virus; HCV, hepatitis C virus; ALD, alcohol-associated liver disease; AIH, autoimmune hepatitis; NAFLD, non-alcoholic fatty liver disease; PBC/PSC, primary biliary cholangitis/primary sclerosing cholangitis; HCC, hepatocellular carcinoma

REFERENCES

1. Lim MS, Goh GBB, Chang JPE, et al. A study of 3013 cases of hepatocellular carcinoma: Etiology and therapy before and during the current decade. *JGH Open* 2021; **5**(9): 1015-8.
2. Lin BZ, Lin TJ, Lin CL, et al. Differentiation of clinical patterns and survival outcomes of hepatocellular carcinoma on hepatitis B and nonalcoholic fatty liver disease. *Journal of the Chinese Medical Association* 2021; **84**(6): 606-13.
3. Pinyopornpanish K, Al-Yaman W, Dasarathy S, Romero-Marrero C, McCullough A. Hepatocellular Carcinoma in Patients Without Cirrhosis: The Fibrosis Stage Distribution, Characteristics and Survival. *Digestive Diseases & Sciences* 2021; **23**: 23.
4. Safcak D, Drazilova S, Gazda J, et al. Nonalcoholic Fatty Liver Disease-Related Hepatocellular Carcinoma: Clinical Patterns, Outcomes, and Prognostic Factors for Overall Survival-A Retrospective Analysis of a Slovak Cohort. *Journal of Clinical Medicine* 2021; **10**(14): 20.
5. Jung YB, Yoo JE, Han DH, et al. Clinical and survival outcomes after hepatectomy in patients with non-alcoholic fatty liver and hepatitis B-related hepatocellular carcinoma. *HPB* 2021; **23**(7): 1113-22.
6. Hiraoka A, Kumada T, Tada T, et al. Efficacy of lenvatinib for unresectable hepatocellular carcinoma based on background liver disease etiology: multi-center retrospective study. *Sci Rep* 2021; **11**(1): 16663.
7. Kumar R, Goh BBG, Kam JW, Chang P, Tan CK. Comparisons between non-alcoholic steatohepatitis and alcohol-related hepatocellular carcinoma. *Clinical and Molecular Hepatology* 2020; **26**(2): 196-208.
8. Paik JM, Golabi P, Biswas R, Alqahtani S, Venkatesan C, Younossi ZM. Nonalcoholic Fatty Liver Disease and Alcoholic Liver Disease are Major Drivers of Liver Mortality in the United States. *Hepatology Communications* 2020; **4**(6): 890-903.
9. Ahn SY, Kim SB, Song IH. Clinical Patterns and Outcome of Hepatocellular Carcinoma in Patients with Nonalcoholic Fatty Liver Disease. *Canadian Journal of Gastroenterology & Hepatology* 2020; **2020**: 4873875.
10. Hanumanthappa N, Cho BH, McKay A, et al. Epidemiology, clinical treatment patterns, and survival of hepatocellular carcinoma in Manitoba. *Canadian Liver Journal* 2020; **3**(2): 194-202.
11. Wang S, Toy M, Hang Pham TT, So S. Causes and trends in liver disease and hepatocellular carcinoma among men and women who received liver transplants in the U.S., 2010-2019. *PLoS One* 2020; **15**(9): e0239393.
12. Liew ZH, Goh GB, Hao Y, Chang PE, Tan CK. Comparison of Hepatocellular Carcinoma in Patients with Cryptogenic Versus Hepatitis B Etiology: A Study of 1079 Cases Over 3 Decades. *Digestive Diseases & Sciences* 2019; **64**(2): 585-90.
13. Tateishi R, Uchino K, Fujiwara N, et al. A nationwide survey on non-B, non-C hepatocellular carcinoma in Japan: 2011-2015 update. *Journal of Gastroenterology* 2019; **54**(4): 367-76.
14. Bengtsson B, Stal P, Wahlin S, Bjorkstrom NK, Hagstrom H. Characteristics and outcome of hepatocellular carcinoma in patients with NAFLD without cirrhosis. *Liver International* 2019; **39**(6): 1098-108.
15. Patkar S, Parray A, Mahendra B, Kurunkar S, Goel M. Performance of Hong Kong Liver Cancer staging system in patients of hepatocellular carcinoma treated with surgical resection: An Indian validation study. *Journal of Surgical Oncology* 2019; **120**(7): 1119-25.
16. Hester CA, Rich NE, Singal AG, Yopp AC. Comparative Analysis of Nonalcoholic Steatohepatitis- Versus Viral Hepatitis- and Alcohol-Related Liver Disease-Related Hepatocellular Carcinoma. *J Natl Compr Canc Netw* 2019; **17**(4): 322-9.
17. Pinero F, Costa P, Boteon YL, et al. A changing etiologic scenario in liver transplantation for hepatocellular carcinoma in a multicenter cohort study from Latin America. *Clinics & Research in Hepatology & Gastroenterology* 2018; **42**(5): 443-52.
18. Pinero F, Pages J, Marciano S, et al. Fatty liver disease, an emerging etiology of hepatocellular carcinoma in Argentina. *World Journal of Hepatology* 2018; **10**(1): 41-50.

19. Ioannou GN, Green P, Lowy E, Mun EJ, Berry K. Differences in hepatocellular carcinoma risk, predictors and trends over time according to etiology of cirrhosis. *PLoS One* 2018; **13**(9): e0204412.
20. Kim NG, Nguyen PP, Dang H, et al. Temporal trends in disease presentation and survival of patients with hepatocellular carcinoma: A real-world experience from 1998 to 2015. *Cancer* 2018; **124**(12): 2588-98.
21. Sadler EM, Mehta N, Bhat M, et al. Liver Transplantation for NASH-Related Hepatocellular Carcinoma Versus Non-NASH Etiologies of Hepatocellular Carcinoma. *Transplantation* 2018; **102**(4): 640-7.
22. Yoon CH, Jin YJ, Lee JW. Nonalcoholic fatty liver disease-associated hepatocellular carcinoma in a hepatitis B virus-endemic area. *Eur J Gastroenterol Hepatol* 2018; **30**(9): 1090-6.
23. Marot A, Henrion J, Knebel JF, Moreno C, Deltenre P. Alcoholic liver disease confers a worse prognosis than HCV infection and nonalcoholic fatty liver disease among patients with cirrhosis: An observational study. *PLoS ONE* 2017; **12**(10).
24. Kimura T, Kobayashi A, Tanaka N, et al. Clinicopathological characteristics of non-B non-C hepatocellular carcinoma without past hepatitis B virus infection. *Hepatology Research* 2017; **47**(5): 405-18.
25. Pais R, Fartoux L, Goumard C, et al. Temporal trends, clinical patterns and outcomes of NAFLD-related HCC in patients undergoing liver resection over a 20-year period. *Aliment Pharmacol Ther* 2017; **46**(9): 856-63.
26. Than NN, Ghazanfar A, Hodson J, et al. Comparing clinical presentations, treatments and outcomes of hepatocellular carcinoma due to hepatitis C and non-alcoholic fatty liver disease. *Qjm* 2017; **110**(2): 73-81.
27. Piscaglia F, Svegliati-Baroni G, Barchetti A, et al. Clinical patterns of hepatocellular carcinoma in nonalcoholic fatty liver disease: A multicenter prospective study. *Hepatology* 2016; **63**(3): 827-38.
28. van Meer S, van Erpecum KJ, Sprengers D, et al. Hepatocellular carcinoma in cirrhotic versus noncirrhotic livers: results from a large cohort in the Netherlands. *European Journal of Gastroenterology & Hepatology* 2016; **28**(3): 352-9.
29. Lopes Fde L, Coelho FF, Kruger JA, et al. INFLUENCE OF HEPATOCELLULAR CARCINOMA ETIOLOGY IN THE SURVIVAL AFTER RESECTION. *Arq Bras Cir Dig* 2016; **29**(2): 105-8.
30. Mittal S, Sada YH, El-Serag HB, et al. Temporal trends of nonalcoholic fatty liver disease-related hepatocellular carcinoma in the veteran affairs population. *Clinical Gastroenterology & Hepatology* 2015; **13**(3): 594-601.e1.
31. Younossi ZM, Otgonsuren M, Henry L, et al. Association of nonalcoholic fatty liver disease (NAFLD) with hepatocellular carcinoma (HCC) in the United States from 2004 to 2009. *Hepatology* 2015; **62**(6): 1723-30.
32. Beste LA, Leipertz SL, Green PK, Dominitz JA, Ross D, Ioannou GN. Trends in burden of cirrhosis and hepatocellular carcinoma by underlying liver disease in US veterans, 2001-2013. *Gastroenterology* 2015; **149**(6): 1471-82.e5; quiz e17-8.
33. Tateishi R, Okanoue T, Fujiwara N, et al. Clinical characteristics, treatment, and prognosis of non-B, non-C hepatocellular carcinoma: a large retrospective multicenter cohort study. *J Gastroenterol* 2015; **50**(3): 350-60.
34. Dyson J, Jaques B, Chattopadyhay D, et al. Hepatocellular cancer: the impact of obesity, type 2 diabetes and a multidisciplinary team. *Journal of hepatology* 2014; **60**(1): 110-7.
35. Paranagua-Vezozzo DC, Ono SK, Alvarado-Mora MV, et al. Epidemiology of HCC in Brazil: incidence and risk factors in a ten-year cohort. *Annals of Hepatology* 2014; **13**(4): 386-93.
36. Weinmann A, Koch S, Niederle IM, et al. Trends in epidemiology, treatment, and survival of hepatocellular carcinoma patients between 1998 and 2009: an analysis of 1066 cases of a German HCC Registry. *J Clin Gastroenterol* 2014; **48**(3): 279-89.
37. Teixeira AC, Mente ED, Cantao CAB, et al. Clinical characteristics of 130 patients with hepatocellular carcinoma followed at a tertiary hospital from Brazil. *World Journal of Oncology* 2012; **3**(4): 165-72.

38. Arase Y, Kobayashi M, Suzuki F, et al. Difference in malignancies of chronic liver disease due to non-alcoholic fatty liver disease or hepatitis C in Japanese elderly patients. *Hepatology Research* 2012; **42**(3): 264-72.
39. Hernandez-Alejandro R, Croome KP, Drage M, et al. A comparison of survival and pathologic features of non-alcoholic steatohepatitis and hepatitis C virus patients with hepatocellular carcinoma. *World Journal of Gastroenterology* 2012; **18**(31): 4145-9.
40. Reddy SK, Steel JL, Chen HW, et al. Outcomes of curative treatment for hepatocellular cancer in nonalcoholic steatohepatitis versus hepatitis C and alcoholic liver disease. *Hepatology* 2012; **55**(6): 1809-19.
41. Hucke F, Sieghart W, Schöniger-Hekele M, Peck-Radosavljevic M, Müller C. Clinical characteristics of patients with hepatocellular carcinoma in Austria - is there a need for a structured screening program? *Wien Klin Wochenschr* 2011; **123**(17-18): 542-51.
42. Ascha MS, Hanouneh IA, Lopez R, Tamimi TA, Feldstein AF, Zein NN. The incidence and risk factors of hepatocellular carcinoma in patients with nonalcoholic steatohepatitis. *Hepatology* 2010; **51**(6): 1972-8.
43. Tokushige K, Hashimoto E, Yatsuji S, et al. Prospective study of hepatocellular carcinoma in nonalcoholic steatohepatitis in comparison with hepatocellular carcinoma caused by chronic hepatitis C. *J Gastroenterol* 2010; **45**(9): 960-7.
44. Sanyal AJ, Banas C Fau - Sargeant C, Sargeant C Fau - Luketic VA, et al. Similarities and differences in outcomes of cirrhosis due to nonalcoholic steatohepatitis and hepatitis C. (0270-9139 (Print)).
45. Hashimoto E, Taniai M, Kaneda H, et al. Comparison of hepatocellular carcinoma patients with alcoholic liver disease and nonalcoholic steatohepatitis. *Alcoholism: Clinical & Experimental Research* 2004; **28**(8 Suppl Proceedings): 164S-8S.
46. Malik SM, Gupte PA, de Vera ME, Ahmad J. Liver transplantation in patients with nonalcoholic steatohepatitis-related hepatocellular carcinoma. *Clin Gastroenterol Hepatol* 2009; **7**(7): 800-6.
47. Amarapurkar DN, Dharod M, Gautam S, Patel N. Risk of development of hepatocellular carcinoma in patients with NASH-related cirrhosis. *Tropical Gastroenterology* 2013; **34**(3): 159-63.
48. Jain D, Nayak NC, Saigal S. Hepatocellular carcinoma in nonalcoholic fatty liver cirrhosis and alcoholic cirrhosis: risk factor analysis in liver transplant recipients. *Eur J Gastroenterol Hepatol* 2012; **24**(7): 840-8.
49. Ertle J, Dechêne A, Sowa J-P, et al. Non-alcoholic fatty liver disease progresses to hepatocellular carcinoma in the absence of apparent cirrhosis. *International Journal of Cancer* 2011; **128**(10): 2436-43.
50. Schütte K, Schulz C, Poranzke J, et al. Characterization and prognosis of patients with hepatocellular carcinoma (HCC) in the non-cirrhotic liver. *BMC Gastroenterol* 2014; **14**: 117.
51. Koh YX, Tan HJ, Liew YX, et al. Liver Resection for Nonalcoholic Fatty Liver Disease-Associated Hepatocellular Carcinoma. *J Am Coll Surg* 2019; **229**(5): 467-78.e1.
52. Yang JD, Hainaut P, Gores GJ, Amadou A, Plymoth A, Roberts LR. A global view of hepatocellular carcinoma: trends, risk, prevention and management. *Nature reviews Gastroenterology & hepatology* 2019; **16**(10): 589-604.
53. Wakai T, Shirai Y, Sakata J, Korita PV, Ajioka Y, Hatakeyama K. Surgical outcomes for hepatocellular carcinoma in nonalcoholic fatty liver disease. *J Gastrointest Surg* 2011; **15**(8): 1450-8.
54. Morisco F, Guarino M, Valvano MR, et al. Metabolic disorders across hepatocellular carcinoma in Italy. *Liver Int* 2018; **38**(11): 2028-39.
55. Gawrieh S, Dakhoul L, Miller E, et al. Characteristics, aetiologies and trends of hepatocellular carcinoma in patients without cirrhosis: a United States multicentre study. *Aliment Pharmacol Ther* 2019; **50**(7): 809-21.
56. D'Silva M, Na HY, Cho JY, et al. Pathological prognostic factors for post-resection survival in patients with hepatocellular carcinoma associated with non-alcoholic fatty liver disease. *Translational Cancer Research* 2021; **10**(7): 3345-55.
57. Rimini M, Kudo M, Tada T, et al. Nonalcoholic steatohepatitis in hepatocarcinoma: new insights about its prognostic role in patients treated with lenvatinib. *ESMO Open* 2021; **6**(6): 100330.

58. Haldar D, Kern B, Hodson J, et al. Outcomes of liver transplantation for non-alcoholic steatohepatitis: A European Liver Transplant Registry study. *Journal of hepatology* 2019; **71**(2): 313-22.
59. Kalaitzakis E, Gunnarsdottir SA, Josefsson A, Björnsson E. Increased risk for malignant neoplasms among patients with cirrhosis. *Clin Gastroenterol Hepatol* 2011; **9**(2): 168-74.
60. Nilsson E, Anderson H, Sargenti K, Lindgren S, Prytz H. Clinical course and mortality by etiology of liver cirrhosis in Sweden: a population based, long-term follow-up study of 1317 patients. *Aliment Pharmacol Ther* 2019; **49**(11): 1421-30.
61. Tohra S, Duseja A, Taneja S, et al. Experience With Changing Etiology and Nontransplant Curative Treatment Modalities for Hepatocellular Carcinoma in a Real-Life Setting—A Retrospective Descriptive Analysis. *Journal of Clinical and Experimental Hepatology* 2021; **11**(6): 682-90.

Supplementary Material 3: Proportion of NAFLD-related hepatocellular carcinoma across time periods and by study setting

	No. of studies	No. of patients	Pooled Proportion	Confidence Interval	Cochran-Q	I^2	p value*
Overall analysis							**0.045**
Overall	42	86311	15.07	11.88 - 18.93	<0.01	99.40%	
Before 2000	1	440	9.77	7.33 - 12.92	-	-	
2000 - 2004	6	4036	9.79	4.34 - 20.58	<0.01	96.70%	
2005 - 2009	21	54822	15.60	10.89 - 21.85	<0.01	99.10%	
2010 and beyond	14	27013	16.97	12.17 - 23.16	<0.01	98.90%	
Western Pacific							**<0.001**
Overall	7	7880	16.69	10.56 - 25.37	<0.01	98.20%	
Before 2000	Insufficient data						
2000 - 2004	1	2180	27.34	25.51 - 29.25	-	-	
2005 - 2009	2	1618	11.93	8.19 - 17.05	<0.01	92.40%	
2010 and beyond	4	4082	17.26	8.59 - 31.68	<0.01	98.60%	
Southeast Asia							-
Overall	3	936	21.94	12.59 - 35.41	<0.01	91.80%	
Before 2000	Insufficient data						
2000 - 2004	Insufficient data						
2005 - 2009	Insufficient data						
2010 and beyond	3	936	21.94	12.59 - 35.41	<0.01	91.80%	
Europe							0.058
Overall	14	26039	15.52	11.50 - 20.62	<0.01	98.60%	
Before 2000	1	440	9.77	7.33 - 12.92	-	-	
2000 - 2004	3	1340	13.67	6.91 - 25.26	<0.01	95.50%	
2005 - 2009	8	22599	17.00	10.85 - 25.63	<0.01	99.00%	
2010 and beyond	2	1930	14.46	12.96 - 16.10	0.89	0.00%	
Americas							**<0.001**
Overall	18	51186	13.23	8.19 - 20.68	<0.01	99.90%	
Before 2000	Insufficient data						
2000 - 2004	2	516	3.49	2.21 - 5.47	0.34	0.00%	

2005 - 2009	11	30605	15.27	8.40 - 26.14	<0.01	99.10%	
2010 and beyond	5	20065	15.42	8.04 - 27.56	<0.01	99.20%	
Subgroup analysis for the Americas							
North America							**<0.001**
Overall	12	49388	17.22	10.46 - 27.01	<0.01	99.70%	
Before 2000	Insufficient data						
2000 - 2004	1	447	3.80	2.38 - 6.03	-	-	
2005 - 2009	8	29979	18.20	10.15 - 30.69	<0.01	99.40%	
2010 and beyond	3	19162	21.90	10.52 - 40.08	<0.01	99.40%	
South America							0.156
Overall	6	1598	6.94	2.66 - 16.92	0.01	83.60%	
Before 2000	Insufficient data						
2000 - 2004	1	69	1.45	0.20 - 9.58	-	-	
2005 - 2009	3	626	8.67	1.75 33.56	<0.01	88.20%	
2010 and beyond	2	903	9.21	5.94 - 14.02	0.03	77.70%	
Subgroup analysis for study setting							**p value****
Clinical cohort	31	22569	14.82	11.08 - 19.55	<0.01	97.60%	0.793
Administrative database	11	63742	15.81	10.61 - 22.89	<0.01	99.80%	

* = p value refers to the subgroup difference for changes in the proportion of NAFLD-related HCC across time periods, in each geographical region
** = p value refers to subgroup difference for proportion of NAFLD-related HCC in clinical cohort studies versus administrative database studies

Supplementary Material 4: Comparison of patient characteristics in NAFLD-related HCC versus HCC from other etiologies; stratified by etiology

	No. of Studies	No. of patients	Effect Size	95% Confidence Interval	P-Value	Cochran-Q	I^2 (%)
**Hepatitis B**							
Age	11	11993	MD: 8.00	6.12 to 9.88	**<0.001**	<0.01	87.7
Body Mass Index	7	3227	MD: 2.62	0.56 to 4.68	**0.013**	<0.01	95.9
Male Gender	13	12236	OR: 0.53	0.37 to 0.75	**0.002**	<0.01	69.6
Diabetes	9	9742	OR: 2.68	1.17 to 6.16	**0.026**	<0.01	93.6
Hypertension	6	8293	OR: 3.46	1.32 to 9.11	**0.021**	<0.01	89.4
Hyperlipidaemia	1	179	OR: 2.58	1.41 to 4.73	**0.002**	-	-
Cardiovascular Disease	2	6190	OR: 1.81	0.02 to 132.47	0.330	<0.01	92.7
Cirrhosis	8	2830	OR: 0.30	0.16 to 0.60	**0.004**	<0.01	69.2
**Hepatitis C**							
Age	11	31923	MD: 5.58	3.70 to 7.45	**<0.001**	<0.01	97.1
Body Mass Index	8	16272	MD: 3.64	2.05 to 5.23	**<0.001**	<0.01	98.4
Male Gender	14	32288	OR: 0.79	0.53 to 1.17	0.212	<0.01	83.2
Diabetes	10	28405	OR: 6.28	3.25 to 12.13	**<0.001**	<0.01	97.4
Hypertension	6	13380	OR: 2.97	1.07 to 8.22	**0.040**	<0.01	94.7
Hyperlipidaemia	2	901	OR: 4.07	0.00 to 127000	0.335	0.05	74.7
Cardiovascular Disease	4	15716	OR: 2.36	0.83 to 6.71	0.080	<0.01	95.9
Cirrhosis	7	3623	OR: 0.16	0.06 to 0.44	**0.004**	<0.01	88.2
**Alcohol**							
Age	13	13794	MD: 4.74	3.80 to 5.89	**<0.001**	<0.01	78.8
Body Mass Index	9	4968	MD: 2.54	1.88 to 3.19	**<0.001**	<0.01	80.9
Male Gender	14	14108	OR: 0.17	0.11 to 0.28	**<0.001**	<0.01	84.1
Diabetes	12	12276	OR: 2.75	1.55 to 4.87	**<0.001**	<0.01	94.0
Hypertension	9	8310	OR: 2.27	1.31 to 3.93	**<0.001**	<0.01	86.7
Hyperlipidaemia	4	463	OR: 2.70	1.19 to 6.10	**0.031**	0.26	24.5
Cardiovascular Disease	3	6863	OR: 2.40	0.33 to 17.7	0.201	<0.01	97.2
Cirrhosis	9	3486	OR: 0.32	0.17 to 0.60	**0.003**	0.01	58.9

Supplementary Material 4B: Comparison of patient and tumor characteristics in NAFLD-related HCC versus HBV-related HCC; by geographical region

Versus hepatitis B	No. of Studies	No. of patients	Effect Size	95% Confidence Interval	P-Value	Cochran-Q	I^2 (%)	Subgroup difference*
Age								0.526
Western Pacific	6	4354	8.82	7.17 to 10.47	<0.001	0.13	41.7	
Europe	1	65	8.80	3.76 to 13.84	<0.001	-	-	
Americas	4	7574	6.95	4.12 to 9.78	<0.001	<0.01	93.5	
Body Mass Index								0.166
Western Pacific	4	1792	1.57	0.55 to 2.59	0.003	0.2	35.5	
Europe	2	230	4.20	0.59 to 7.82	0.023	<0.01	91.1	
Americas	1	1205	2.70	1.70 to 3.70	<0.001	-	-	
Male Gender								0.791
Western Pacific	7	4432	0.51	0.35 - 0.76	0.006	0.15	35.2	
Europe	2	230	0.71	0.29 - 1.71	0.798	0.14	53.2	
Americas	4	7574	0.46	0.13 - 1.62	0.143	<0.01	87.1	
Diabetes								0.846
Western Pacific	5	3275	3.27	1.75 - 6.10	<0.001	0.09	50.1	
Europe	1	65	3.36	1.21 - 9.36	0.006	-	-	
Americas	3	6402	1.37	1.10 - 1.72	0.020	<0.01	97.8	
Hypertension								0.808
Western Pacific	4	3096	3.19	1.47 - 6.94	<0.001	0.07	57.3	
Europe	Insufficient data							
Americas	2	5197	1.79	1.34 - 2.40	<0.001	<0.01	96.8	
Cirrhosis								**0.017**
Western Pacific	5	2421	0.23	0.12 - 0.45	0.003	0.15	41.2	
Europe	2	230	0.33	0.14 - 0.75	0.009	0.03	80	
Americas	1	179	0.90	0.40 - 2.02	0.324	-	-	
Tumor diameter								**<0.001**
Western Pacific	5	3275	1.14	0.68 to 1.61	<0.001	0.41	0.2	
Europe	1	165	-1.00	-1.44 to -0.56	<0.001	-	-	
Americas	1	179	-0.60	-2.10 to 0.90	0.432	-	-	

*=Subgroup difference refers to comparison of effect size between geographical region

Supplementary Material 4C: Comparison of patient and tumor characteristics in NAFLD-related HCC versus HCV-related HCC; by geographical region

Versus hepatitis C	No. of Studies	No. of patients	Effect Size	95% Confidence Interval	P-Value	Cochran-Q	I^2 (%)	Subgroup difference*
Age								**<0.001**
Western Pacific	2	290	-0.40	-2.89 to 2.09	0.754	0.32	0.0	
Europe	3	1314	4.09	-6.34 to 14.52	0.442	<0.01	98.6	
Americas	6	29186	7.77	6.13 to 9.41	<0.001	<0.01	96.1	
Body Mass Index								**0.002**
Western Pacific	2	290	1.01	-0.44 to 2.45	0.171	0.14	54.4	
Europe	4	1515	4.50	1.81 to 7.19	0.001	<0.01	97.9	
Americas	2	14467	4.29	2.92 to 5.66	<0.001	<0.01	95.0	
Male Gender								**0.004**
Western Pacific	3	454	0.98	0.39 - 2.46	0.944	0.43	0.0	
Europe	4	1515	1.50	0.68 - 3.29	0.200	0.03	66.1	
Americas	7	30319	0.52	0.32 - 0.86	0.020	<0.01	81.7	
Diabetes								0.663
Western Pacific	2	290	1.86	1.14 - 3.02	0.014	<0.01	93.3	
Europe	3	1314	8.03	4.74 - 13.59	0.003	0.48	0.0	
Americas	5	26801	7.99	2.62 - 24.41	0.007	<0.01	98.7	
Hypertension								**<0.001**
Western Pacific	2	290	1.37	0.84 - 2.26	0.203	0.68	0.0	
Europe	1	756	5.42	3.62 - 8.13	<0.001	-	-	
Americas	3	12334	1.33	1.16 - 1.52	<0.001	<0.01	95.7	
Cirrhosis								**<0.001**
Western Pacific	1	164	0.84	0.31 - 2.31	0.741	-	-	
Europe	4	1515	0.06	0.02 - 0.16	<0.001	0.16	41.6	
Americas	2	1944	0.27	0.19 - 0.38	<0.001	0.24	26.5	
Tumor diameter								0.118
Western Pacific	2	290	1.40	-1.89 to 4.68	0.405	<0.01	95.1	
Europe	2	957	1.11	0.63 to 1.58	<0.001	0.05	74.6	
Americas	2	892	-0.13	-1.22 to 0.95	0.811	0.17	47.3	

*=Subgroup difference refers to comparison of effect size between geographical region

Supplementary Material 4D: Comparison of patient and tumor characteristics in NAFLD-related HCC versus alcohol-associated HCC; by geographical region

Versus alcohol	No. of Studies	No. of patients	Effect Size	95% Confidence Interval	P-Value	Cochran-Q	I^2 (%)	Subgroup difference*
Age								**0.008**
Western Pacific	6		4.17	2.22 to 6.11	<0.001	0.03	59.0	
Europe	1	55	2.70	-1.05 to 6.48	0.159	-	-	
Americas	5	10600	4.60	3.50 to 5.7	<0.001	<0.01	85.1	
Body Mass Index								0.675
Western Pacific	5		2.25	0.80 to 3.71	0.028	0.03	65.5	
Europe	2	369	2.77	1.11 to 4.43	0.001	0.19	40.9	
Americas	2	3898	2.65	1.37 to 3.92	<0.001	<0.01	91.3	
Male Gender								**0.001**
Western Pacific	6	2720	0.10	0.08 - 0.12	<0.001	0.82	0	
Europe	2	369	0.33	0.17 - 0.62	<0.001	0.22	34.9	
Americas	6	11006	0.24	0.12 - 0.50	0.004	<0.01	78.8	
Diabetes								0.460
Western Pacific	6	2720	2.49	1.01 - 6.13	0.048	0.01	75.0	
Europe	1	55	1.29	0.41 - 4.01	0.664	-	-	
Americas	5	9488	3.19	0.90 - 11.23	0.063	<0.01	97.4	
Hypertension								0.698
Western Pacific	6	2720	2.12	1.76 - 2.55	<0.001	0.70	0	
Europe	Insufficient data							
Americas	3	5590	2.80	0.13 - 59.94	0.285	<0.01	94.7	
Cirrhosis								**0.020**
Western Pacific	5	2466	0.35	0.18 - 0.70	0.013	0.17	38.0	
Europe	2	369	0.02	0.01 - 0.11	<0.001	0.26	21.9	
Americas	2	651	0.47	0.32 - 0.70	<0.001	0.22	34.7	
Tumor diameter								0.413
Western Pacific	5	2621	1.03	-0.38 to 2.43	0.152	<0.01	87.5	
Europe	1	314	0.1	0.03 to 0.17	0.003	-	-	
Americas	1	245	0.3	-0.91 to 1.51	0.627	-	-	

*=Subgroup difference refers to comparison of effect size between geographical region

Supplementary Material 5: Comparison of tumor characteristics in NAFLD-related HCC versus HCC from other etiologies; stratified by etiology

	No. of Studies	No. of patients	Effect Size	95% Confidence Interval	P-Value	Cochran-Q	I^2 (%)
**Hepatitis B**							
Tumour Diameter	7	3619	MD: 0.52	-0.48 to 1.52	0.310	<0.01	87.5
Alpha-fetoprotein	6	2883	MD -0.19	-0.42 to 0.04	0.109	<0.01	72.7
Uni-nodular HCC	6	4379	OR 1.23	0.97 - 1.56	0.079	0.66	0.00%
BCLC 0/A	4	2639	OR 1.02	0.55 - 1.90	0.913	0.16	42.10%
BCLC B	5	2818	OR 1.12	0.74 - 1.72	0.488	0.43	0.00%
BCLC C/D	5	2818	OR 0.80	0.39 - 1.63	0.433	0.04	59.90%
**Hepatitis C**							
Tumour Diameter	6	2139	MD: 0.78	0.07 to 1.48	0.031	<0.01	87.3
Alpha-fetoprotein	8	10709	MD 0.02	-0.09 to 0.13	0.744	0.06	49.0
Uni-nodular HCC	3	454	OR 0.81	0.11 - 5.86	0.697	0.05	66.20%
BCLC 0/A	2	956	OR 0.69	0.31 - 1.51	0.104	0.7	0.00%
BCLC B	3	1767	OR 1.25	0.67 - 2.33	0.257	0.48	0.00%
BCLC C/D	3	1767	OR 1.10	0.33 - 3.68	0.766	0.02	73.70%
**Alcohol**							
Tumour Diameter	7	3180	MD: 0.56	0.12 to 1.00	**0.012**	<0.01	81.4
Alpha-fetoprotein	7	2939	MD: 0.19	-0.04 to 0.41	**0.100**	<0.01	75.5
Uni-nodular HCC	4	2430	OR 1.12	0.60 - 2.08	0.612	0.07	57.70%
BCLC 0/A	3	582	OR 0.79	0.16 - 3.89	0.591	0.07	61.50%
BCLC B	4	827	OR 1.37	1.07 - 1.76	0.028	0.94	0.00%
BCLC C/D	4	827	OR 1.11	0.36 - 3.43	0.783	<0.01	75.60%

Abbreviations: BCLC = Barcelona Clinic Liver Cancer ; ECOG = Eastern Cooperative Oncology Group; BCLC: Barcelona Clinic Liver Cancer

Supplementary Material 6: Factors associated with receiving curative therapy* among patients with NAFLD-related HCC

Factors	No. of studies	No. of patients	Odds Ratio	95% Confidence Interval	P-Value
Age	18	30082	0.54	0.39 to 0.74	**<0.001**
Male	18	30082	1.04	0.95 to 1.13	0.438
Body Mass Index	13	25236	1.19	0.59 to 2.39	0.633
Diabetes	14	7463	0.96	0.89 to 1.03	0.270
Hypertension	11	6429	0.96	0.89 to 1.07	0.390
Hyperlipidaemia	6	3186	0.97	0.90 to 1.04	0.421
Cirrhosis	16	8808	0.93	0.89 to 0.98	**0.004**
BCLC 0/A	7	5716	1.04	1.02 to 1.06	**<0.001**
BCLC C/D	7	5716	0.96	0.94 to 0.98	**<0.001**
Tumour diameter	11	4873	0.92	0.41 to 2.06	0.839
Alpha-fetoprotein	12	7129	1.00	1.00 to 1.00	0.639

Legend: *defined as liver transplantation, liver resection or ablation

Abbreviations: BCLC = Barcelona Clinic Liver Cancer ; ECOG = Eastern Cooperative Oncology Group

Supplementary Material 7: Summary of risk factors affecting overall survival (OS) and disease-free survival (DFS) of NAFLD-related HCC

	No. of Studies	Beta-Coefficient	95% Confidence Interval	P-Value
**Overall survival**				
Age	21	-0.01	-0.03 to 0.01	0.540
Male Gender	22	-0.24	-1.20 to 0.72	0.623
Body Mass Index	14	0.01	-0.03 to 0.05	0.605
Diabetes	16	0.01	-1.10 to 1.11	0.990
Hypertension	12	0.05	-0.64 to 0.74	0.886
Hyperlipidaemia	5	0.74	-1.20 to 2.69	0.454
Cardiovascular Disease	4	0.41	-2.07 to 2.88	0.746
Early BCLC stage	10	0.26	-0.54 to 1.07	0.517
Late BCLC stage	10	0.09	-0.92 to 1.10	0.863
Tumour Diameter	12	0.01	-0.10 to 0.13	0.836
Alpha-fetoprotein	12	0.00	0.00 to 0.00	0.918
**Disease free survival**				
Age	8	0.01	-0.03 to 0.05	0.653
Male Gender	9	-0.89	-3.73 to 1.95	0.540
Body Mass Index	4	-0.08	-0.19 to 0.03	0.164
Diabetes	5	-0.39	-2.05 to 1.28	0.651
Hypertension	5	-0.44	-1.59 to 0.70	0.448
Tumour Diameter	7	0.03	-0.15 to 0.21	0.719
Alpha-fetoprotein	4	-0.02	-0.03 to 0.00	**0.029**

Legend: *denote statistical significance at p<0.05; BCLC: Barcelona Clinic Liver Cancer

Supplementary Material 8: Overall and disease-free survival in NAFLD-related HCC vs HCC from other etiologies; stratified by individual etiology

	No. of Studies	No. of Patients	Hazard Ratio	95% Confidence Interval	P-Value	Cochran-Q	I^2 (%)	Subgroup difference**
**Overall survival**								
Hepatitis C virus	9	3997	1.24	0.86 to 1.81	0.253	<0.01	99.8	0.388
Western Pacific	1	90	0.90	0.49 to 1.66	0.746	-	-	
Europe	5	1921	1.42	0.88 to 2.31	0.150	<0.01	99.7	
Americas	3	1986	0.94	0.78 to 1.14	0.938	0.14	49.9	
Hepatitis B virus	10	4790	1.10	0.88 to 1.38	0.384	<0.01	97.7	0.573
Western Pacific	5	2918	0.92	0.70 to 1.21	0.531	0.61	0.0	
Europe	2	595	1.26	0.86 to 1.84	0.238	<0.01	99.7	
Americas	2	198	1.45	0.35 to 6.05	0.614	<0.01	94.1	
South-East Asia	1	1079	1.05	0.87 to 1.28	0.596	-	-	
Alcohol	8	1933	1.19	0.94 to 1.52	0.148	<0.01	88.1	0.299
Western Pacific	1	229	0.93	0.67 to 1.28	0.653	-	-	
Europe	3	933	1.10	0.80 to 1.53	0.559	<0.01	91.5	
Americas	3	672	1.71	0.82 to 3.55	0.150	<0.01	92.0	
South-East Asia	1	99	0.78	0.49 to 1.25	0.303	-	-	
**Disease-free survival**								

Hepatitis C Virus	5	700	0.67	0.31 to 1.42	0.286	<0.01	79.9	
Hepatitis B Virus	5	2135	1.02	0.61 to 1.72	0.942	0.70	0.0	0.226
Western Pacific	3	1793	0.80	0.55 to 1.17	0.244	0.16	0.0	
Europe	1	323	1.24	0.77 to 1.97	0.378	-	-	
Americas	1	19	0.48	0.12 to 1.84	0.281	-	-	
Alcoholic Liver Disease	2	344	0.92	0.69 to 1.23	0.571	0.43	0.00	
**Curative therapy Only***								
**Overall survival**								
Hepatitis C Virus	2	1243	1.22	1.01 to 1.48	**0.041**	0.97	0.00	
Hepatitis B Virus	2	1715	1.01	0.61 to 1.67	0.955	0.78	0.00	

Legend: *defined as liver transplantation, liver resection and ablation;
**subgroup difference refers to comparison of effect sizes between geographical regions for each etiology

Abbreviations: NAFLD, non-alcoholic fatty liver disease; hepatocellular carcinoma, HCC

Supplementary Material 9: Funnel plots for publication bias

Funnel plot of age between NAFLD and non-NAFLD-related HCC

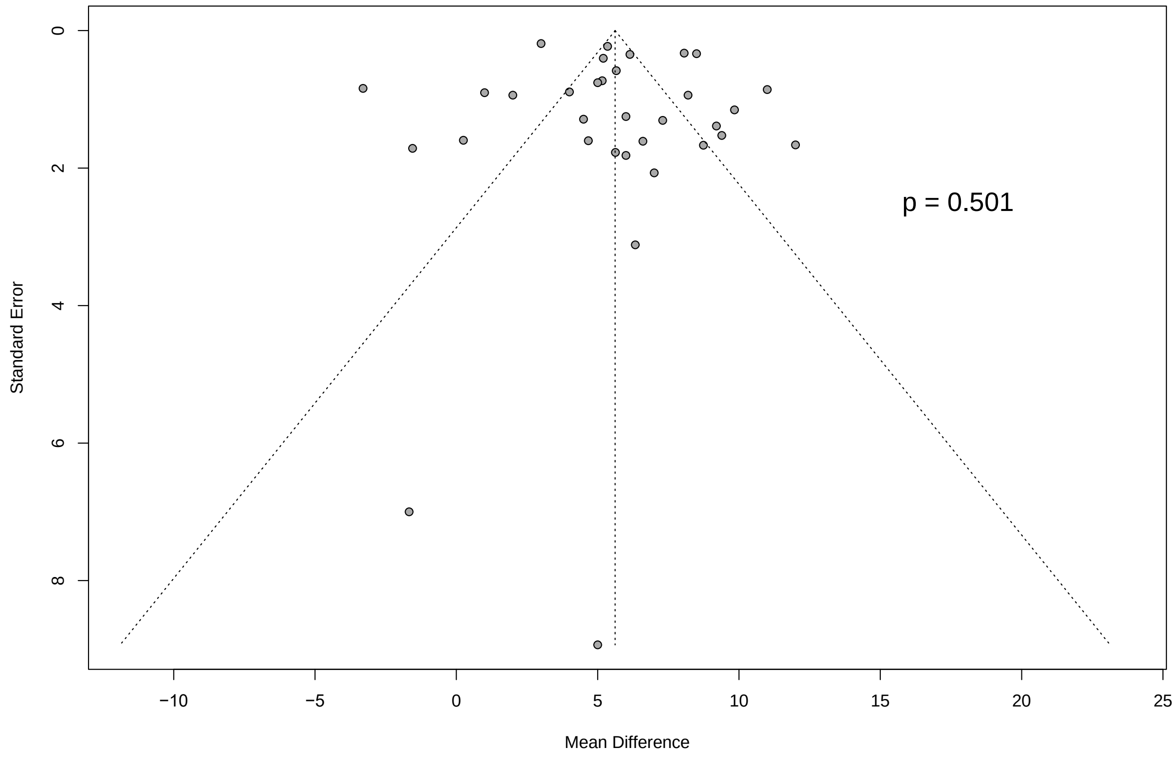

Funnel plot of body mass index (BMI) between NAFLD and non-NAFLD-related HCC

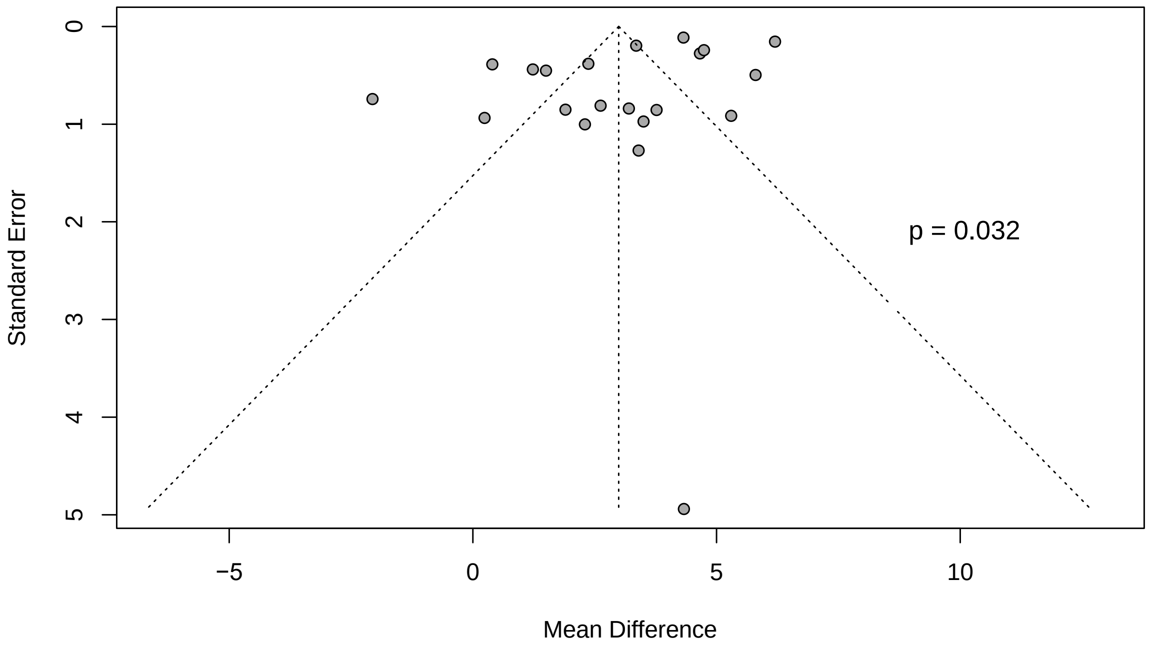

Funnel plot of diabetes between NAFLD and non-NAFLD-related HCC

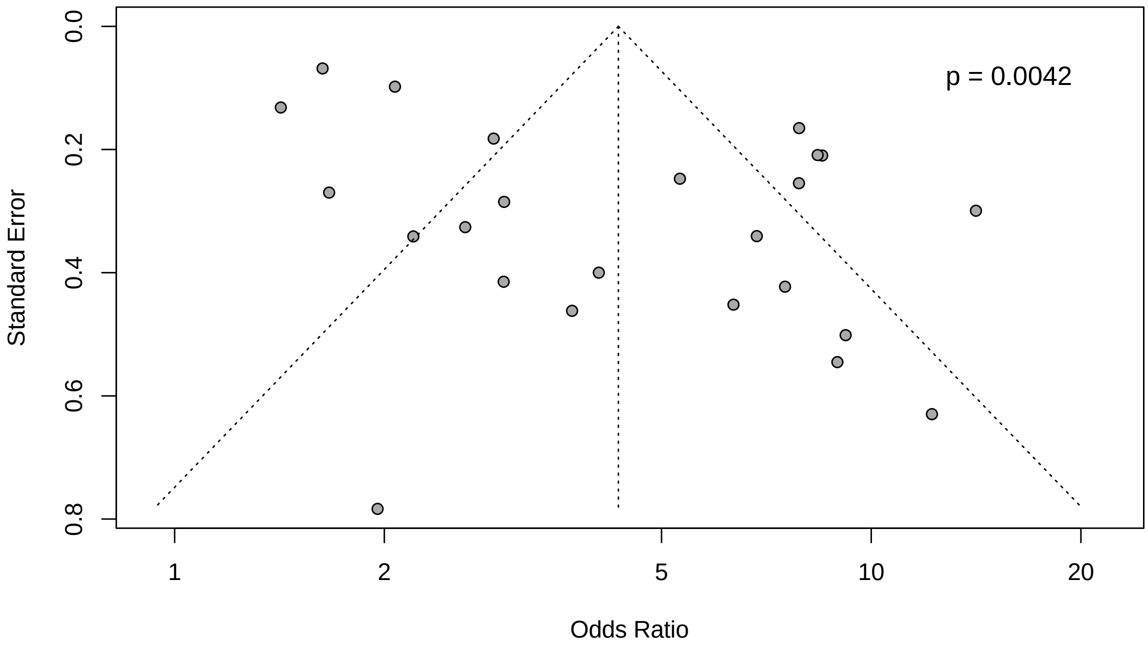

Funnel plot of hypertension between NAFLD and non-NAFLD-related HCC

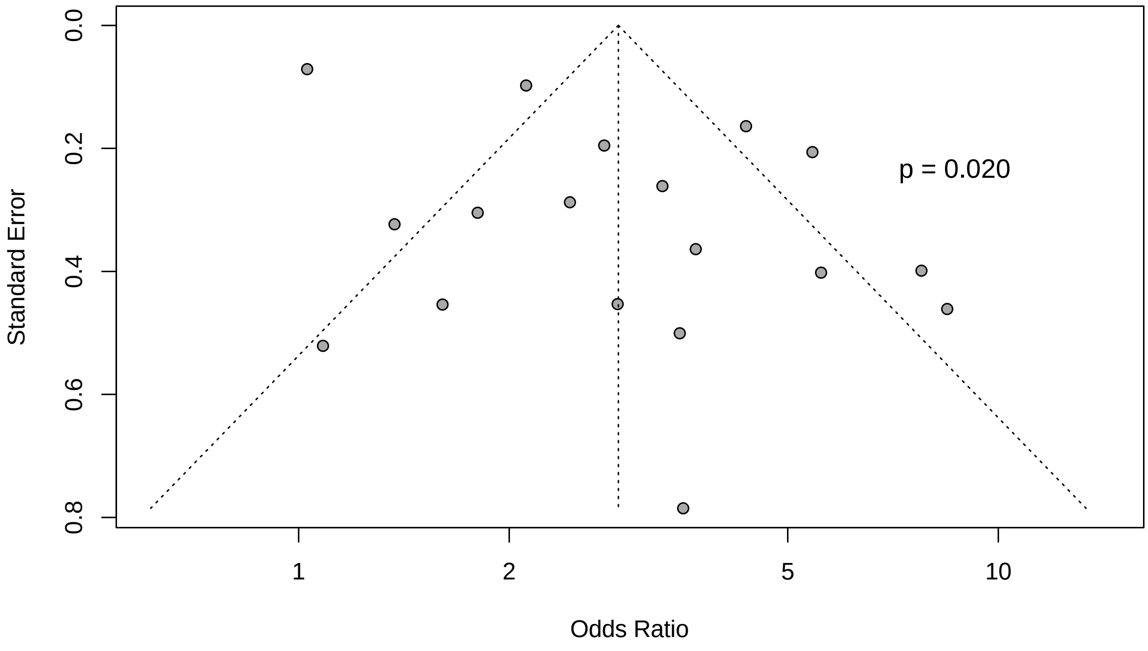

Funnel plot of male gender between NAFLD and non-NAFLD-related HCC

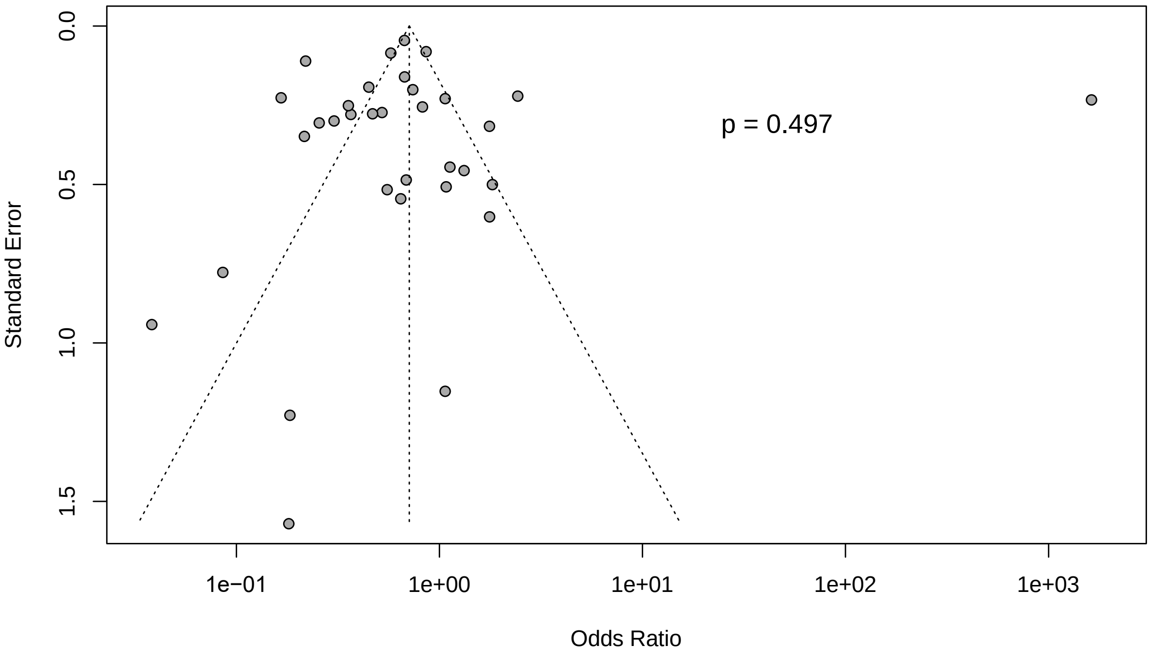

Funnel plot of non-cirrhosis between NAFLD and non-NAFLD-related HCC

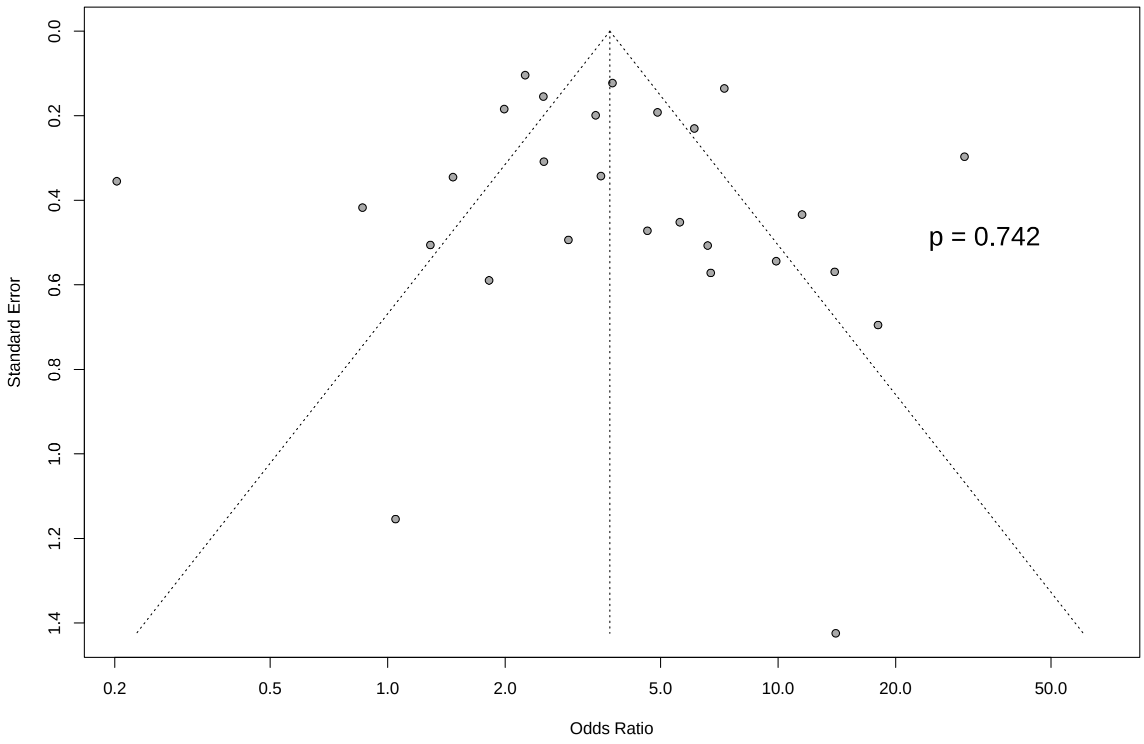

Funnel plot of mean tumor diameter between NAFLD and non-NAFLD-related HCC

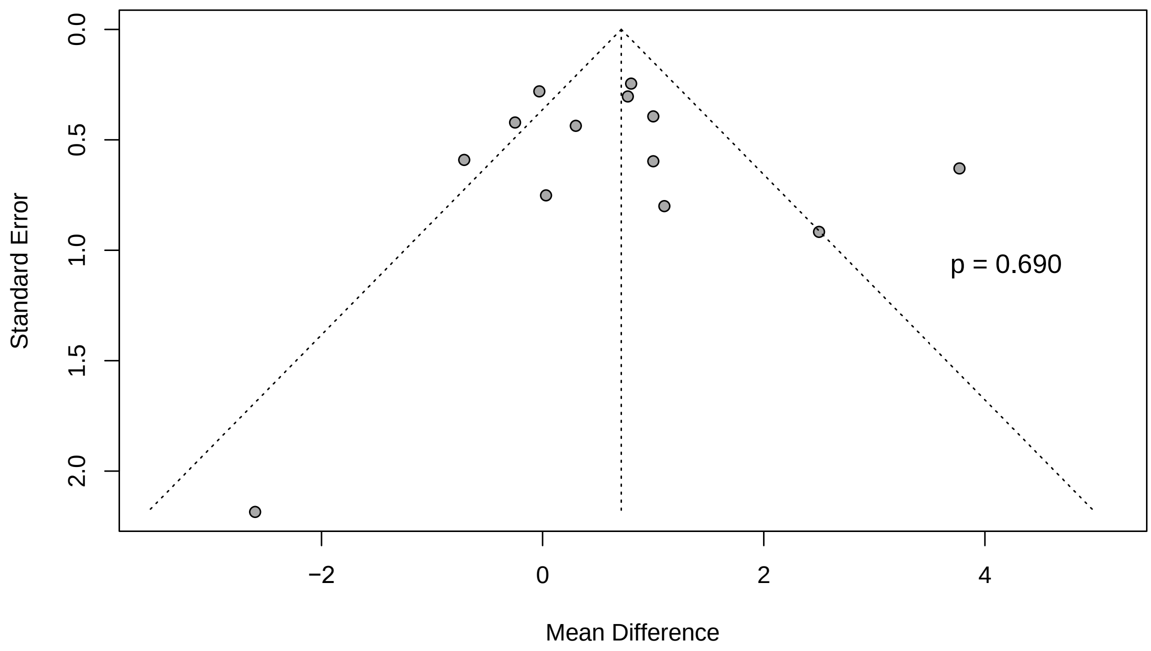

Funnel plot of unimodular tumor between NAFLD and non-NAFLD-related HCC

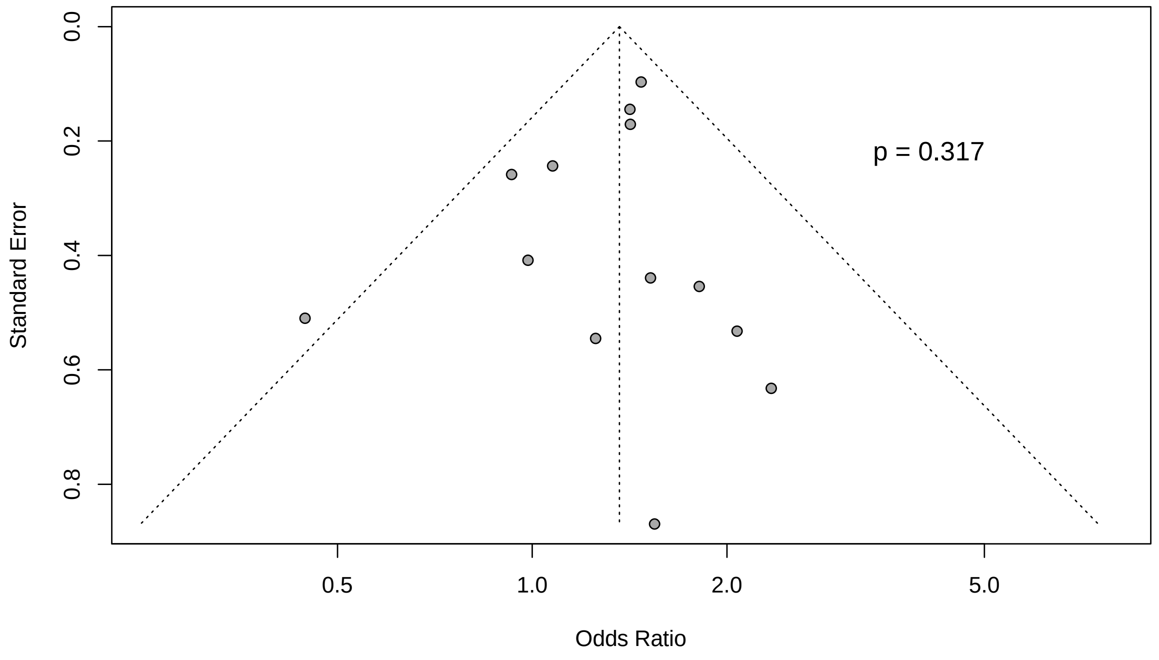

Funnel plot of BCLC B between NAFLD and non-NAFLD-related HCC

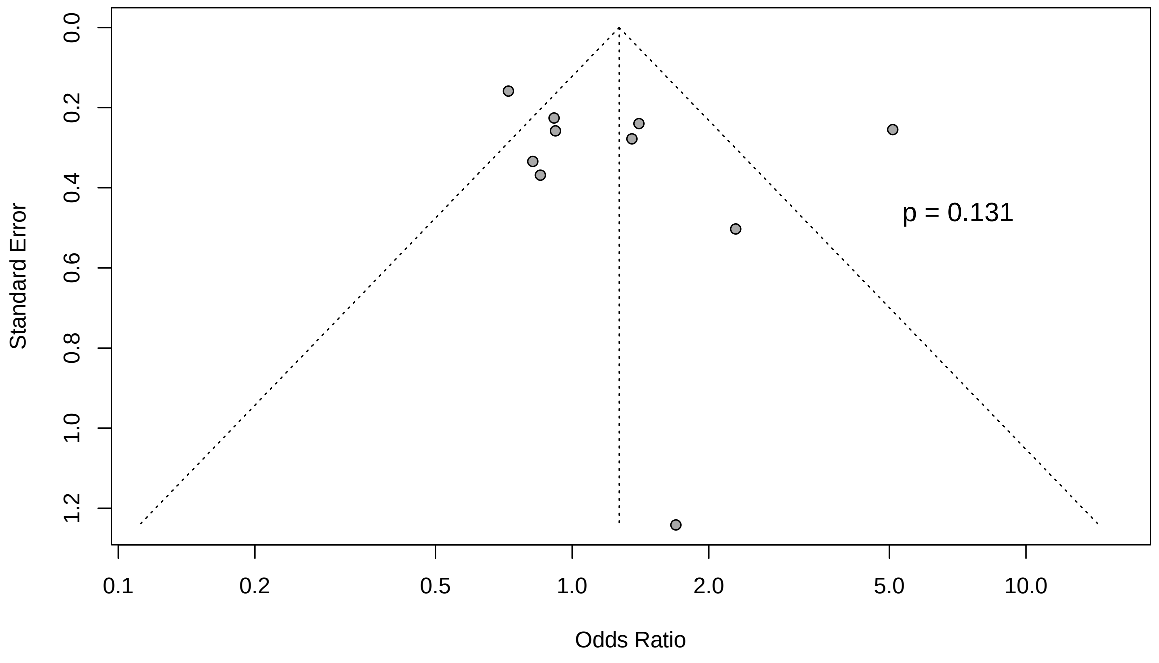

Funnel plot of BCLC C/D between NAFLD and non-NAFLD-related HCC

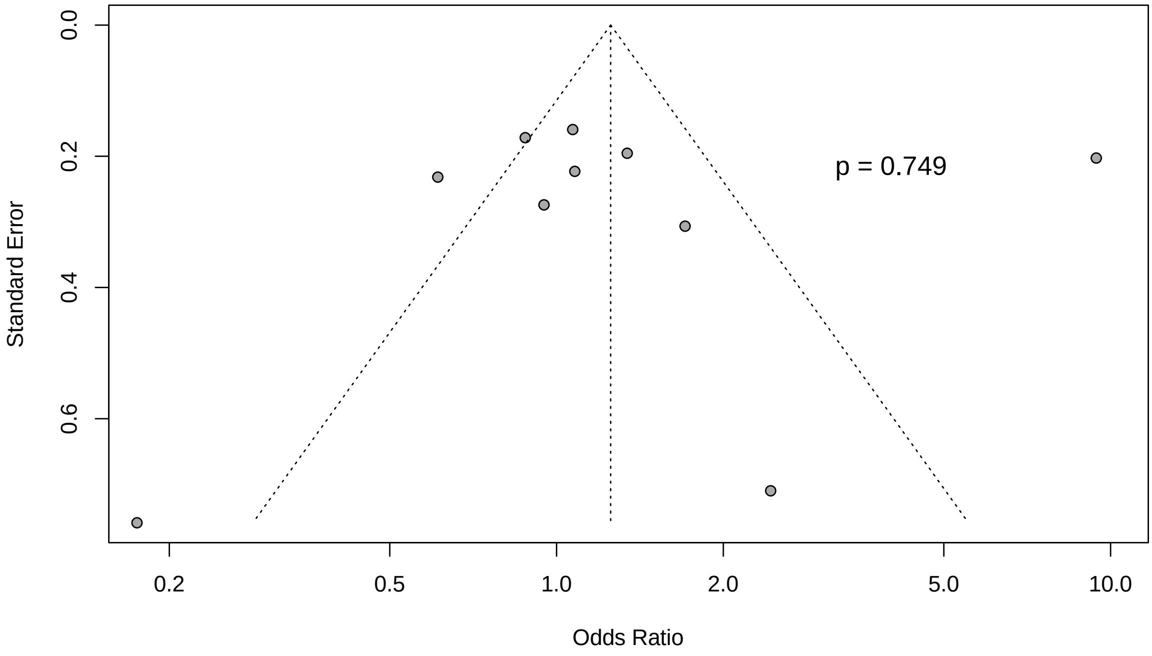

Funnel plot of allocation of curative treatment between NAFLD and non-NAFLD-related HCC

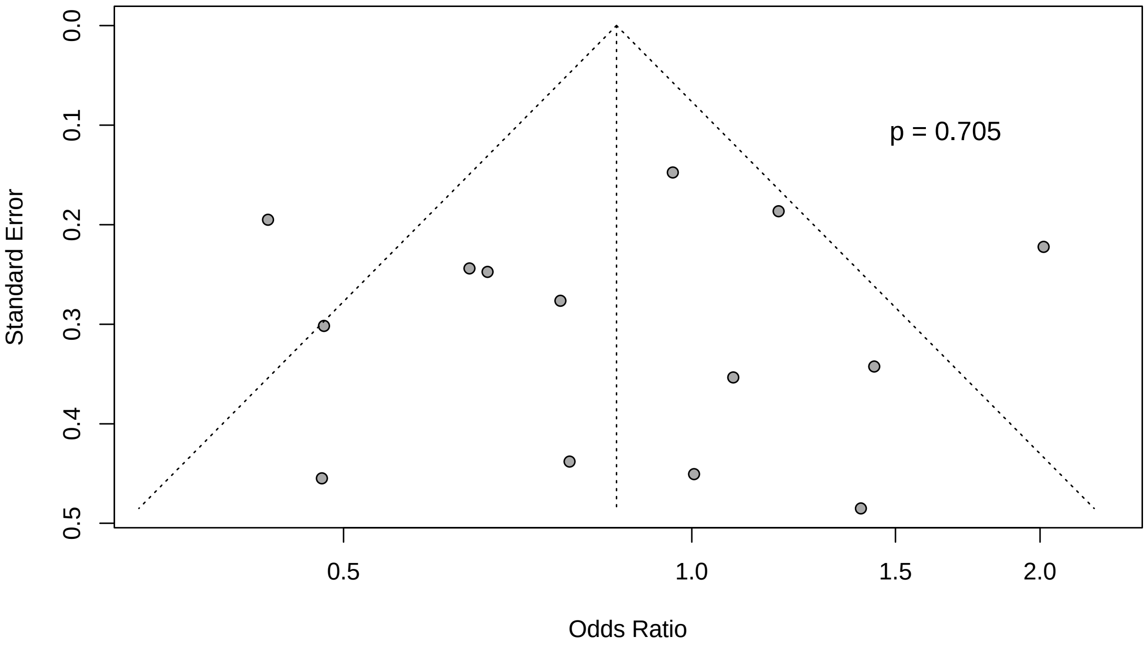

Funnel plot of allocation of palliative treatment between NAFLD and non-NAFLD-related HCC

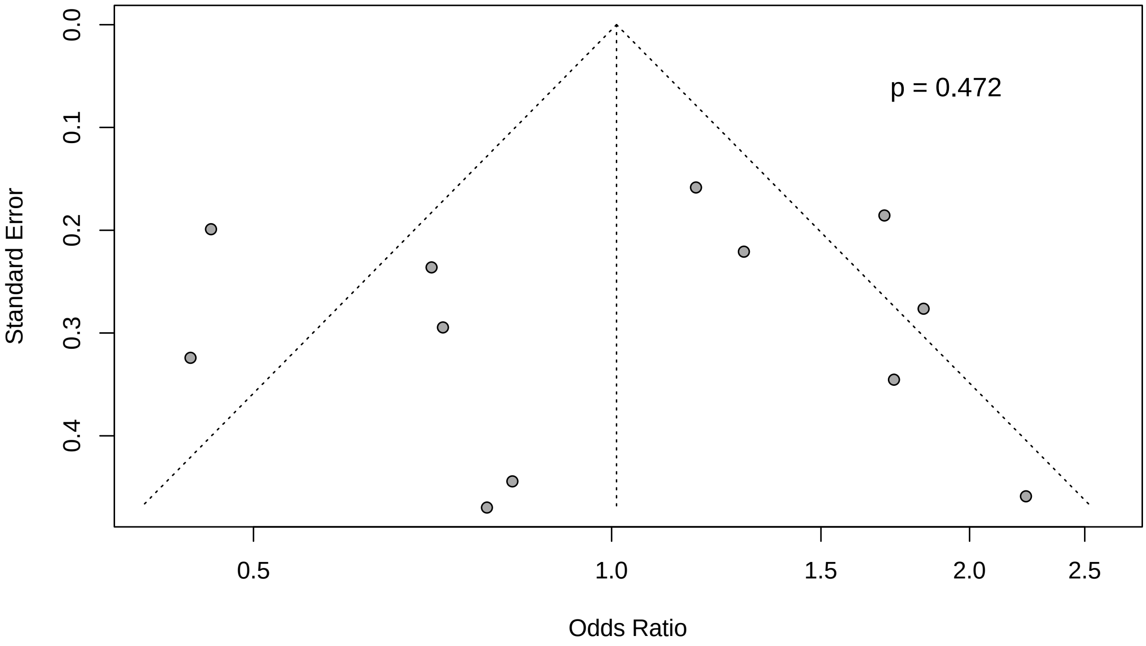

Funnel plot of overall survival (OS) between NAFLD and non-NAFLD-related HCC

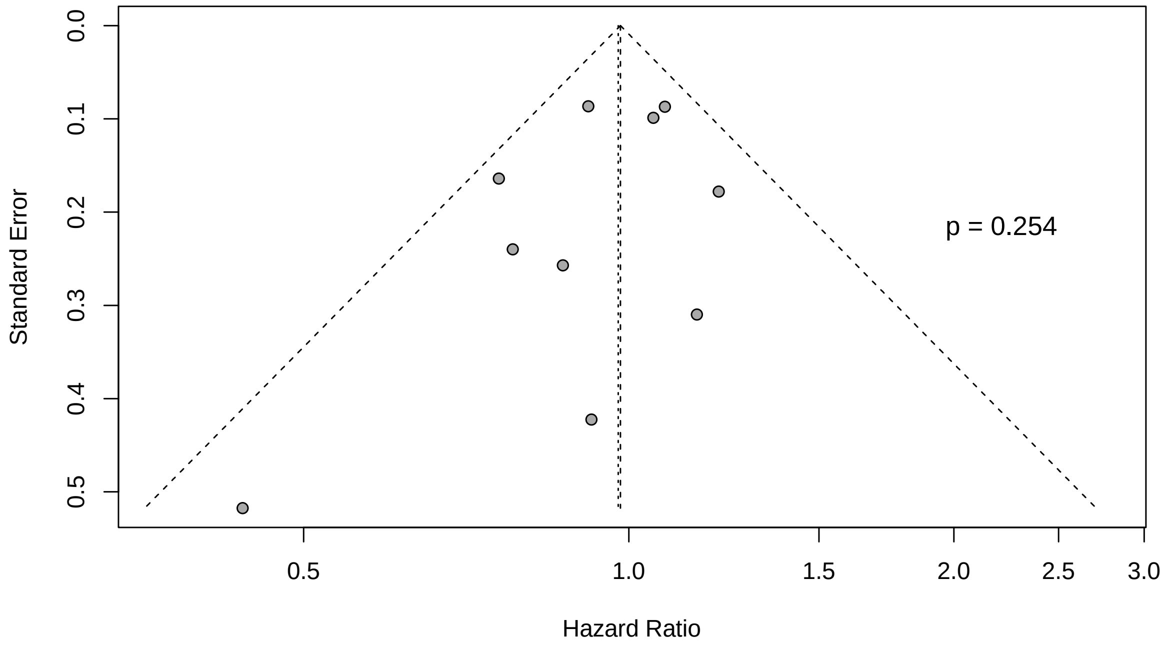

Funnel plot of disease-free survival (DFS) between NAFLD and non-NAFLD-related

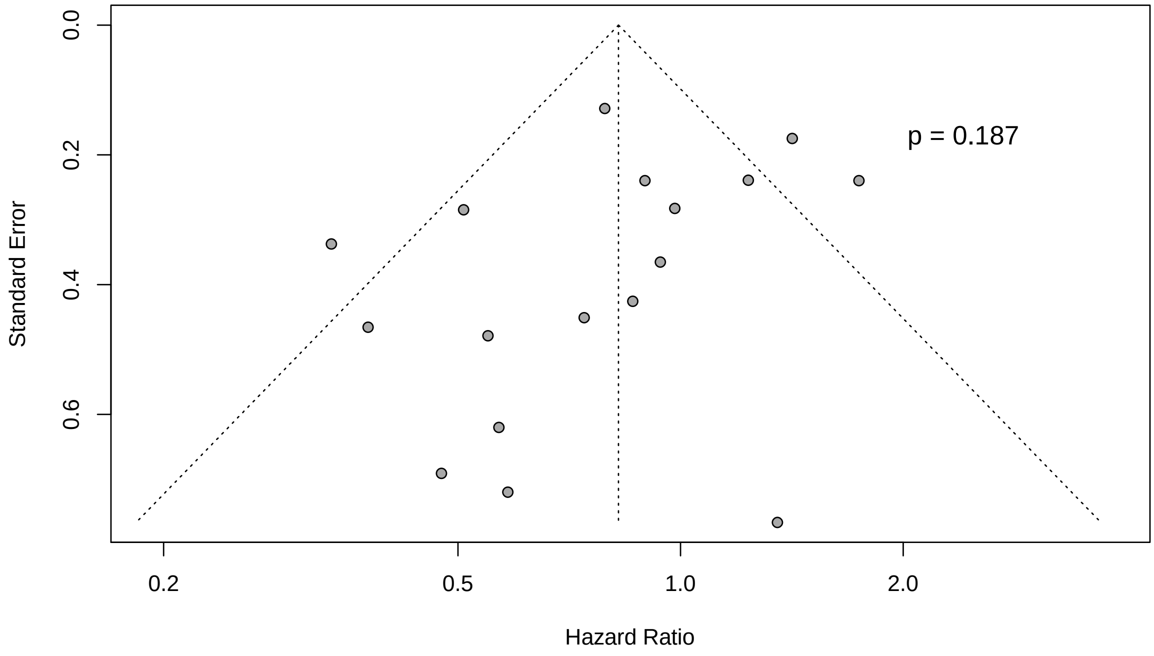

NAFLD HCC Meta-analysis Protocol

Background:

Non-alcoholic fatty liver disease (NAFLD) is the fastest rising cause of hepatocellular carcinoma (HCC) in the U.S. and parts of Europe, and is expected to rise exponentially in parallel with the global obesity epidemic.[1-5] However, the characteristics and outcomes of NAFLD HCC versus HCC from other etiologies, including hepatitis B (HBV), hepatitis C (HBV), and alcoholic liver disease (ALD), remain unclear. Existing studies have reported contrasting results, in part due to these studies being limited by geographical region or treatment.[6-8]

Therefore, we aim to conduct a systematic review and meta-analysis to compare the clinical features, prevalence, surveillance rates and outcomes of NAFLD-related HCC versus non-NAFLD HCC.

Aims:

(1) To determine the prevalence of HCC secondary to NAFLD, globally, by region, and over time

(2) To evaluate the differences in patient characteristics (e.g. age, gender, presence of metabolic conditions including diabetes, hypertension, hyperlipidemia, presence of cirrhosis) and tumor characteristics (e.g. tumor number, tumor diameter, BCLC staging) and surveillance between NAFLD HCC and other etiologies, overall and by etiology

(3) To determine differences in treatment allocation (curative treatment, palliative treatment, best supportive care) between NAFLD HCC and other etiologies, overall and by etiology

(4) To determine differences in survival outcomes (overall survival, disease-free survival) between NAFLD HCC and other etiologies, overall and by etiology

Inclusion and exclusion criteria:

Only studies written or translated to English language will be included, with no date filter. Prospective and retrospective cohort studies and randomized control trials will be considered for inclusion. Studies will be included if they (1) describe the prevalence of HCC secondary to NAFLD, the patient and tumor characteristic of NAFLD HCC, and the treatment allocation and survival outcomes of NAFLD HCC; and (2) compared these outcomes with HCC secondary to other etiologies (HBV, HCV, ALD). Studies that diagnosed NAFLD based on either (i) imaging, (ii) histology or (iii) ICD codes in the absence of

significant alcohol consumption and coexisting causes of chronic liver disease will be included for analysis.

Studies that were not published will be excluded. Editorials, case series/ report, review articles will also be excluded. Studies will be excluded if they included patients with HCC secondary to ‘cryptogenic’ causes.

Analysis:

A meta-analysis of proportions will be conducted using a generalized linear mixed model with Clopper-Pearson intervals to determine the prevalence of HCC secondary to NAFLD. Subgroup analysis will be conducted to determine the proportion of HCC secondary to NAFLD by geographical region according to the WHO regions, and by time period.

Comparative meta-analysis will be conducted in odds ratio and weighted mean difference to compare between patient characteristics, tumor characteristics, and treatment allocation between NAFLD HCC versus other etiologies. Subgroup analysis will be conducted for comparative outcomes stratified by individual etiology of HCC (i.e. HBV, HCV, ALD). Survival outcomes between NAFLD HCC and other etiologies will be performed via pooled analysis of hazard ratios. Subgroup analysis for survival outcomes will be stratified by presence of cirrhosis, and by type of treatment received. All comparative analysis will be conducted using the DerSimonian-Laird random effects model. All analysis will be conducted in R Studio using the ‘meta’ package. Quality assessment of included articles will done via the Joanna Briggs Institute (JBI) Critical Appraisal Tool

REFERENCES

1. Younossi Z, Stepanova M, Ong JP, et al. Nonalcoholic Steatohepatitis Is the Fastest Growing Cause of Hepatocellular Carcinoma in Liver Transplant Candidates. *Clin Gastroenterol Hepatol*; **17**(4): 748-55.e3.
2. Dyson J, Jaques B, Chattopadyhay D, et al. Hepatocellular cancer: the impact of obesity, type 2 diabetes and a multidisciplinary team. *Journal of hepatology* 2014; **60**(1): 110-7.
3. Estes C, Chan HLY, Chien RN, et al. Modelling NAFLD disease burden in four Asian regions-2019-2030. *Aliment Pharmacol Ther*; **51**(8): 801-11.
4. Estes C, Razavi H, Loomba R, Younossi Z, Sanyal AJ. Modeling the epidemic of nonalcoholic fatty liver disease demonstrates an exponential increase in burden of disease. *Hepatology* 2018; **67**(1): 123-33.
5. Huang DQ, El-Serag HB, Loomba R. Global epidemiology of NAFLD-related HCC: trends, predictions, risk factors and prevention. *Nat Rev Gastroenterol Hepatol* 2021; **18**(4): 223-38.
6. Wong CR, Nguyen MH, Lim JK. Hepatocellular carcinoma in patients with non-alcoholic fatty liver disease. *World journal of gastroenterology* 2016; **22**(37): 8294.
7. Younossi ZM, Otgonsuren M, Henry L, et al. Association of nonalcoholic fatty liver disease (NAFLD) with hepatocellular carcinoma (HCC) in the United States from 2004 to 2009. *Hepatology* 2015; **62**(6): 1723-30.
8. Benhammou JN, Aby ES, Shirvanian G, Manansala K, Hussain SK, Tong MJ. Improved survival after treatments of patients with nonalcoholic fatty liver disease associated hepatocellular carcinoma. *Scientific reports* 2020; **10**(1): 1-9.
